# Microneedle loaded with luteolin-colostrum-derived exosomes: a dropless approach for treatment of glaucoma

**DOI:** 10.1007/s13346-025-01914-9

**Published:** 2025-07-11

**Authors:** Sarah A. Elsherbiny, Amal H. El-Kamel, Basant A. Bakr, Lamia A. Heikal

**Affiliations:** 1 Department of Pharmaceutics, Faculty of Pharmacy, Alexandria University, Alexandria, Egypt; 2 Department of Zoology, Faculty of Science, Alexandria University, Alexandria, Egypt

**Keywords:** Microneedles, Extracellular vesicles, Intraocular pressure, Herbal drug, Bio-inspired, Propolis

## Abstract

Glaucoma, a leading cause of irreversible blindness, is marked by elevated intraocular pressure (IOP) and retinal ganglion cell death. Traditional IOP-lowering eye drops often fail to penetrate the ocular barrier, leading to suboptimal outcomes. Microneedles (MN), offer a promising minimally invasive and localized alternative. Our study aimed to formulate a naturally-derived nanodelivery system using Luteolin-loaded colostrum-derived exosomes (LUT-EX) and propolis in MN arrays for better ocular delivery. The isolated exosomes were uniform, averaging 50.83 nm in size, with a zeta potential of -21.89 mV. LUT-EX showed a 48-h sustained release and high safety with an IC50 of 356.3 µg/mL. Integrating LUT-EX and propolis into MN arrays achieved optimal dissolution in over one minute and maintained mechanical strength under 30 N compression. LUT-EX@MN increased LUT permeation through scleral tissues 2.6-fold compared to gel matrix formulations. It also showed a sustained IOP-lowering effect reaching the normal IOP level in the first 3h and sustained over 7 days. The integrated system significantly reversed glaucoma-induced changes in TNF-α, IL-8, MYOC, NRF2, TIMP1, and IL-1β levels, resembling those of the healthy group. It also boosted antioxidant activity, increasing glutathione peroxidase by 1.6-fold compared to glaucomatous rabbits. Thus, our study highlighted that the integration of LUT-EX into microneedle arrays presents a groundbreaking dropless approach for localized glaucoma treatment, offering enhanced therapeutic efficacy. This platform could revolutionize glaucoma management, paving the way for more effective and targeted ocular therapies.

**Graphical Abstract:**

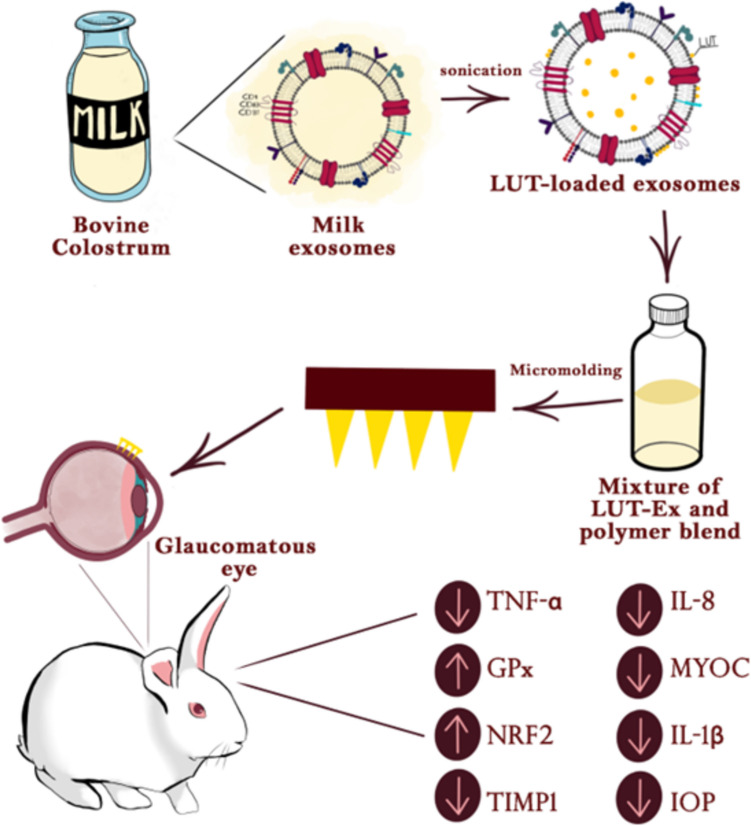

**Supplementary Information:**

The online version contains supplementary material available at 10.1007/s13346-025-01914-9.

## Introduction

Glaucoma is one of the most prevalent ocular diseases that stands as the primary cause of irreversible blindness worldwide. The prevalence of glaucoma is expected to rise to 111.8 million cases by 2040 [1]. Glaucoma refers to a class of eye disorders marked by progressive optic nerve damage, resulting in the degeneration and loss of retinal ganglion cells (RGCs) [2]. Elevated IOP, primarily caused by partial or complete obstruction of the aqueous humor outflow, is a key pathological risk factor in glaucoma. Impaired conventional outflow and elevated IOP can lead to optic nerve damage, potentially progressing to vision loss [3]. While visual field loss in glaucoma is progressive and irreversible, achieving a target IOP within the normal range can slow its progression. The most common anti-glaucoma treatment involves IOP-lowering agents, typically delivered as topical eye drops. However, this approach faces limitations beyond drug efficacy, including short precorneal residence time and reduced drug absorption due to rapid lacrimal fluid turnover, extensive nasolacrimal drainage, and frequent blinking. Furthermore, human-related factors such as poor compliance and lack of persistence, often driven by the need for frequent application, further reduce treatment effectiveness [4]. Owing to these challenges, the compelling necessity for an effective localized targeted drug delivery system has emerged.

Microneedles (MNs) represent one such innovative platform, where targeted drug distribution is made possible by MNs’ ability to pass through the cornea’s and sclera’s barriers, thereby enhancing therapeutic outcomes [5]. Among the various types, dissolving MNs are particularly well-suited for eye diseases as they can be applied like contact lenses, improving patient comfort and compliance. Unlike traditional intraocular injections with hypodermic needles exceeding 10 mm in length, MNs measuring 400–700 microns offer a minimally invasive alternative for ocular drug delivery. This approach reduces discomfort, minimizes tissue damage, and facilitates localized drug administration. Furthermore, dissolving MNs can create drug micro-depots in specific ocular tissues, enabling sustained drug release as the MNs gradually dissolve, making them an ideal platform for extended drug delivery. Furthermore, MNs offer the potential for combination therapy and improved stability, making microneedle-based drug delivery an increasingly attractive approach in the fields of pharmaceuticals and healthcare [5].

Recently, the incorporation of natural bioactive components such as propolis in MNs has gained significant attention for its diverse medicinal properties. Propolis is a resinous substance produced by bees from plant exudates, rich in bioactive compounds, including flavonoids, phenolic acids, and hydrocarbons. It exhibits potent antimicrobial, anti-inflammatory, and antioxidant effects. In addition, recent research has highlighted its neuroprotective potential, particularly in ocular health [6]. Studies suggest that propolis can protect RGCs by modulating apoptosis and inflammatory pathways, making it a promising candidate for preventing neurodegenerative damage in conditions like glaucoma [7]. Moreover, when formulated into nano-preparations, propolis has demonstrated hypotensive effects, further enhancing its therapeutic potential in glaucoma management [8]. These findings underscore its potential role in preserving vision and preventing blindness.

To further exploit the power of nature, utilizing exosomes as innovative natural bioinspired drug delivery systems offers unique yet complementary therapeutic benefits. Exosomes, tiny vesicles secreted by cells, have shown promise as a treatment for glaucoma [9]. Exosomes, a subtype of extracellular vesicles, are generally 30 to 100 nm in diameter and are released into the extracellular environment by various cell types. They are present in a range of biological fluids, including plasma, urine, milk, and amniotic fluid [10]. Milk exosomes have been effectively isolated from various sources, including bovine colostrum, porcine milk, goat milk, and human breast milk. Unlike synthetic nanocarriers, naturally derived nanoparticles like milk exosomes offer several advantages: they are well-tolerated in the body due to their presence in various biological fluids, exhibit cross-species compatibility without triggering immune or inflammatory responses, have a longer circulating half-life and can be readily internalized by other cells [11]. Additionally, colostrum-derived exosomes offer distinct advantages over regular milk exosomes in the treatment of glaucoma, primarily due to their neuroprotective and regenerative properties. Unlike regular milk exosomes, colostrum exosomes are enriched with immune-modulating proteins and growth factors that can help protect and repair RGCs. Their higher concentration of bioactive molecules, including FGF, PDGF, and VEGF, enhances cell survival and regeneration, potentially slowing disease progression and preserving vision. Additionally, colostrum exosomes demonstrate superior drug-loading efficiency, allowing for targeted delivery of glaucoma medications with improved bioavailability and reduced systemic side effects. In contrast, regular milk exosomes, while beneficial, lack the specialized immune and regenerative factors necessary for effective glaucoma management, making colostrum-derived exosomes a more potent therapeutic option [12–14].

Therapeutic compounds can be delivered straight to target cells via these natural messengers of molecules that can cross the blood-retinal barrier. By using exosomes'neuroprotective and regenerative properties, researchers aim to reduce neuroinflammation and stop the loss of RGCs, two key factors in the pathophysiology of glaucoma [9].

It is worth mentioning that herbal drugs are also gaining popularity as complementary treatments for glaucoma. Luteolin (LUT); a naturally occurring flavonoid that may be found in a variety of fruits and vegetables has demonstrated promise in the treatment of glaucoma due to its anti-inflammatory antioxidant, and neuroprotective benefits. LUT is known to reduce both oxidative stress and IOP in the eye, which are crucial for the treatment of glaucoma. It also prevents damage to RGCs by scavenging free radicals [15].

The integration of colostrum-derived exosomes and the natural flavonoid LUT into dissolving MNs containing propolis represents a uniquely synergistic and innovative approach for ocular drug delivery, particularly for treating glaucoma. Unlike conventional MN systems that primarily deliver synthetic drugs, this platform combines the regenerative and immunomodulatory potential of colostrum-derived exosomes—rich in anti-inflammatory miRNAs and growth factors—with the neuroprotective and antioxidant properties of LUT. This dual payload is delivered directly through dissolving MNs, which bypass ocular barriers enabling localized, sustained, and minimally invasive transscleral administration. Moreover, exosomes’ intrinsic targeting capabilities ensure precise delivery to damaged ocular cells, while the MN matrix protects both exosomal and flavonoid components from enzymatic degradation. This triple innovation—biologically sourced exosomes, plant-derived neuroprotectants, and patient-friendly MN delivery—offers a comprehensive, customizable, and scalable platform that surpasses current exosome carriers or MN systems in both therapeutic breadth and translational potential [16, 17].

Therefore, our research aimed to design a nanodelivery system using naturally derived substances such as colostrum-derived exosomes and propolis integrated into MNs for the treatment of glaucoma. The efficacy of the fabricated system in boosting LUT bioavailability in ocular tissues was evaluated. In addition, the feasibility of using the prepared system as a dropless approach for glaucoma treatment was assessed ex vivo and on glaucoma-induced rabbit model in vivo. The safety and effectiveness of the developed formulation were assessed using molecular techniques and laboratory cell culture methods.

## Materials and methods

### Materials

Raw unpasteurized bovine colostrum was purchased from the animal production unit, Faculty of Agriculture, Alexandria University, Egypt, and was used within one hour after collection. Luteolin (LUT) (98% purity) was sourced from Baoji Guokang BioTechnology Co., Ltd, China. Polyvinyl alcohol (PVA) and polyvinyl pyrrolidone (PVP-K90) were obtained from Sigma Aldrich Chemical Co. (UK). Propolis was purchased from Imtenan, Egypt. Bradford and MTT reagents were bought from Sigma Aldrich (UK). PCR primers were obtained from Eurofins Scientific (Luxembourg). A two-step real-time quantitative PCR (RT qPCR) kit (Low ROX- SYBR Green) was purchased from Applied Biotechnology (ABT), Egypt. The rest of the other reagents used were of analytical grade.

### Exosomes isolation from bovine colostrum

Exosomes (EX) were extracted from bovine colostrum milk using the PEG precipitation method. A milk sample of 15 mL was mixed with 25 mL of PBS and subjected to centrifugation at 3000 × g for 15 min at 4 °C to get rid of somatic cells, cellular debris, and milk fat globules. Subsequently, 20 mL of the supernatant was mixed with an equivalent volume of 0.25 M EDTA and incubated on ice for 15 min to precipitate casein and casein-coated exosomes. Afterward, the mixture underwent centrifugation at 12,000 × g for 1 h at 4 °C [18]. The supernatant was then mixed with an equivalent volume of PEG 6000 solution (16% w/w) to achieve a PEG concentration at the end of 8% w/w, and the solution was left at 4 °C overnight. The following day, the mixture was allowed to centrifuge at 4 °C for 30 min at 12,000 × g. After discarding the supernatant, the pellet was reconstituted in 10 mL PBS. Another centrifugation run of the suspension was performed at 4 °C for 30 min at 12,000 × g, after which the supernatant was discarded and the pellet was reconstituted in 10 mL PBS via simple mixing and stored at − 80 °C for future use [19].

### Exosome characterization

#### Particle count, size, and zeta potential

Transmission electron microscopy (TEM) was employed to confirm the morphology as well as the particle size of the isolated exosomes. Samples were diluted 100-fold, applied to copper grids, and stained using uranyl acetate at a concentration of 2% w/v. TEM was performed as an imaging procedure using an electron microscope (JEM-100 CX, JEOL, Tokyo, Japan). Particle size was determined by taking 100 measurements from various fields using Image J analysis software [20] (Fiji 1.52p; National Health Institute, MD, USA). Moreover, the polydispersity index (PDI) was subsequently calculated using the following equation:

$$PDI= {(\frac{SE}{d})}^{2}$$

where SE represents the standard error of deviation, and d denotes the mean diameter.

The colloidal properties of colostrum-derived exosomes were assessed by measuring particle size, zeta potential, and particle count using a direct light scattering technique using Zetasizer (Malvern, Ultra red label, Malvern Panalytical Ltd, UK).

#### Flowcytometry

A modified protocol was applied to analyze the surface tetraspanin proteins (CD9, CD63, and CD81) of colostrum-derived exosomes to confirm the exosomal isolation. Briefly, the exosome sample was vortexed, and 50 μL of magnetic microbeads as a solid support detectable by the flowcytometer were added. This was followed by incubation for one hour at ambient temperature. After incubation, beads bound to exosomes were washed with 1% w/v BSA in PBS, then blocked with PBS containing 10% w/v BSA, and stained using 1 μg/mL of anti-CD9 conjugated with AlexaFluor^®^ 488, anti-CD63 conjugated with AlexaFluor^®^ 647, and anti-CD81 conjugated with AlexaFluor^®^ 546, respectively. The stained samples were incubated at room temperature for one hour, washed, and then resuspended in FACS buffer using the BD FACSCalibur TM flow cytometer (San Jose, USA) for further analysis.

#### Protein content

To measure the protein content of the isolated exosomes, 5 µL of the exosome samples were added to a 96-well plate in triplicate. An aliquot of 250 µL of Bradford reagent was then added to the sample in each well, shielded from light and incubated for 15 min at room temperature, according to the manufacturer’s guidelines for the Bradford reagent. Protein absorbance was measured using a microplate reader (Tecan, infinite F50, Männedorf, Switzerland) set to 595 nm. The concentration was calculated by fitting the absorbance into the regression equation derived from the standard albumin solution’s calibration curve [21].

### Drug loading in exosomes

LUT was loaded into colostrum-derived exosomes using the sonication method. An aliquot of 100 μL of LUT stock solution (3 or 5 mg/mL in methanol) was added to 900 μL of exosome dispersion. The LUT-exosome mixture was subjected to ultrasonication with a Bandelin probe ultrasonic sonicator (Sonoplus, Germany) using a 3 mm diameter probe (MS 73) set at a 20% amplitude. The process consisted of six cycles of 3-s on/off pulses over a total duration of 3 min, with a 3-min resting period on a bath of ice for cooling after each cycle. To allow restoration of the structure of the exosomal membrane, LUT-loaded exosomes (LUT-EX) were incubated for 1 h at 37 °C following sonication [22].

### Entrapment efficiency

The encapsulation efficiency (EE%) of LUT in LUT-EX was calculated by applying a modified version of the method carried out by Aqil et al. [23]. The LUT-EX dispersion was first subjected to centrifugation at 3,000 rpm for 10 min at 4 °C to separate any unentrapped free drug. The collected precipitate was then dissolved in methanol, after which the concentration was analyzed using UV spectrophotometry at 350 nm. To calculate the EE%, the following equation was used:

$$EE\,\%= \frac{Initial\, amount\, of\, LUT-Unentrapped\, LUT }{Initial\, amount\, of\, LUT}\times 100$$

### In vitro drug release

A modified method using dialysis bag was utilized to assess the behavior of LUT release after being loaded in exosomes. LUT-EX and free LUT solution (300 μg/mL in 25% PEG 400) were placed in VISKING^®^ 36/32 dialysis bags (28 mm, MWCO 12,000–14,000; Serva, Heidelberg, Germany) and soaked in 12 mL volume of 25% v/v PEG 400 in PBS (pH 7.4) as a medium for release. This setting was kept at 37 °C with shaking at 100 rpm to ensure sink condition. The samples were collected at specified time points and analyzed spectrophotometrically at 350 nm with the release medium serving as the blank. Withdrawn samples were replaced with fresh release media to sustain sink conditions throughout the experiment. The cumulative amount of LUT released from the exosomes was calculated, corrected, and expressed as a percentage of the total drug released as a function of time [24] using the following equation:

$$\% \,Cumulative\, drug\, release=\frac{Sample\, volume\, taken\, (ml)}{Bath\, volume\, (v)}\times P\left(t-1\right)+Pt$$

where Pt is the percentage of the amount released at the time (t) and P(t-1) is the percentage of the amount released per time (t).

### In vitro studies of cell culture

#### Cell line

Corneal stromal fibroblast cell lines from adult rats (CSF) were isolated at CERRMA (Center of Excellence for Research in Regenerative Medicine and Applications), Faculty of Medicine, Alexandria University. Cell Culture Laboratory, Department of Pharmaceutics, Faculty of Pharmacy, Alexandria University was the site at which the experiments were carried out. CSF cells were cultured in DMEM (High glucose Dulbecco’s modified Eagle’s medium). 10% v/v FBS (Fetal bovine serum) and antibiotics (100 U/mL penicillin and 100 μg/mL streptomycin) were added as supplements to DMEM prior to use. 5 % CO_2_, and 37 ºC were the conditions of the incubator where the cells were maintained.

#### Cytotoxicity assessment

Cellular cytotoxicity of LUT, unloaded exosomes, and LUT-EX were assessed using the MTT assay. 96-well plates were used to seed the CSF cells at a seeding density of 5 × 10^3^ cells/well and left overnight to adhere. Serial dilutions of LUT, unloaded exosomes, and LUT-EX were added and allowed to incubate for 24 h at 37 °C under 5% CO_2_. MTT solution (1 mg/mL) was added after the removal of the medium and left to incubate for a further 3 h. At the end of the experiment, the MTT solution was removed carefully, and the formed purple formazan crystals were dissolved in DMSO. The absorbance of the dissolved purple formazan was measured in an absorbance-based microplate reader (Tecan, Infinite F50, Mannedorf, § Switzerland) at 570 nm. The control, which represents 100% viable cells, was used to normalize the optical density of different sample concentrations. IC_50_ values and cell viability (%) were calculated.

#### Cellular association

The green, fluorescent dye; coumarin 6 (C6) was used to label exosomes to assess their cellular association capability. C6-exosomes were prepared by loading 900 μL of exosomes with 100 μL C6 solution at a concentration of 250 μg/mL in ethanol. A seeding density of 3*10^5^ cells/well was used to seed CSF cells in 6 well plates. They were then incubated for 24 h at 5% CO_2_ and 37 °C for adherence. C6-exosomes and C6 solution were then added to the cells to reach a final concentration of 100 ng/mL in each well and allowed to incubate at 37 °C under 5% CO_2_ for another 4 h or 24 h. Paraformaldehyde at a concentration of 4% v/v (pH 7.4) was used to fix the cells for 10 min in the dark after being washed with phosphate-buffered saline (PBS, pH 7.4) 3 times. The nuclei of the fixed cells were stained using Hoechst 33,342. Confocal laser scanning microscopy (LEICA, DMi8, Mannheim/Wetzlar, Germany) was used to observe the cellular association. Depending on their excitation/emission wavelength, fluorescence signals were measured at 358/416 nm (Hoechst, blue) and 488/530 nm (C6, green). NIH ImageJ software was used to process the images and quantify the intensity of the cell’s fluorescence.

#### Cellular proliferation and migration evaluation using scratch assay

The tissue regeneration experiment via wound healing scratch assay was used to investigate the impact of EX, LUT, and LUT-EX on the regenerative and migratory capability of CSF cells. A seeding density of 5*10^5^ cells/well was used to seed CSF cells in 12-well plates and left to culture until they reached confluency at 37 °C and under 5% CO_2_. To perform this experiment, a single scratch was created in the CSF cell monolayer using a 1 mL pipette tip. A photograph of the formed scratch was then captured using a phase-contrast inverted microscope (CKX41SF; Olympus). The scratched cells were then treated with the tested samples at doses equivalent to half of their IC_50_ values and left to culture in the incubator for an additional 24 h, and the scratch area was then measured. NIH ImageJ software was used to examine the images, and the percentage of the cell-free zone remaining was compared to the initial scratch to calculate the cell migration percentage using the following equation:

$$\%\, Wound\, closure=1-{~}^{At}\!\left/ \!{~}_{A0}\right.$$

*where A*0 = initial wound area and *At* = area at time t.

Migration index and migration rate were also calculated using the following 2 equations respectively: [25]

$$Migration\, index= \frac{Distance\, migrated\, by\, cells}{Initial\, scratch \,width}$$

$$Migration\, rate=\frac{Distance\, migrated\, by\, cells}{Time}$$

### MNs fabrication

MNs were prepared using micro-molding technology with a polymeric matrix composed of 20% w/w PVA, 25% w/w PVP, and 0.5% w/w propolis, based on a previously reported method with minor modifications [26]. For the preparation of blank MNs, a PVA solution was formed by allowing PVA powder to dissolve in deionized water and subjecting it to continuously stir on a magnetic stirrer at 90 °C for 2 h. PVP powder was added to a predetermined volume of water and mixed using a magnetic stirrer. Equal amounts of PVA and PVP gels were combined, and the mixture was centrifuged at a low speed to eliminate air bubbles. Subsequently, 650 µL of the polymer blend and 250 µL of 0.5% propolis were mixed. For the preparation of LUT-loaded MNs (LUT@MN), the polymeric matrix was mixed with a methanolic solution of LUT (3 mg/mL) in a 9:1 w/w ratio. As for drug-free EX-loaded MNs (EX@MN) and LUT-EX-loaded MN (LUT-EX@MN), the PVP powder was added to a predetermined volume of the exosomal dispersion instead of water and then proceeded as mentioned above.

Silicone microneedle molds (H600) purchased from Micropoint Technologies Pte Ltd., Singapore, were used to create conical-shaped dissolving microneedle arrays (10 × 10) with dimensions of 600 µm height and 300 µm width. Approximately 200 mg of the prepared gel mixture was poured into the molds and subjected to centrifugation at 5000 rpm for 15 min to make sure that complete filling of the needle cavities had occurred. The excess gel was carefully removed from the molds, after which a polymeric matrix free of the drug was added to form the backing layer. The molds were then centrifuged again for 5 min. Following this, the MN arrays were left to dry at room temperature in a desiccator for 72 h. Once dried, the MN arrays were carefully extracted from the molds and stored in a sealed container, covered with aluminum foil, and kept refrigerated in a desiccator until further characterization. To verify the integrity of exosomes after being incorporated into MNs, dissolution of the array in deionized water was done, and the aliquots were examined to detect the presence of exosomes using TEM.

### MNs characterization

#### Morphological examination

SM-IT200 Scanning electron microscopy (SEM) (JEOL, Tokyo, Japan) was utilized to examine the shape of MN arrays. A thin layer of gold was used to coat the arrays, and images were captured at 60 × and 250 × magnification.

#### Drug content and drug uniformity

To assess the amount of LUT in the MNs, MN arrays were placed in 2 mL deionized water, vortexed for 10 min, and then subjected to sonication for 15 min. Subsequently, Triton X-100 (0.5% w/v) was added to the solution to destroy the exosomes and release their drug content. This was followed by an additional 15 min sonication in an Elmasonic S 40 H ultrasonication bath (Elma, USA) at 37 kHz frequency. An aliquot (100 μL in volume) was withdrawn, then diluted with methanol up to 1 mL, thoroughly mixed, followed by centrifugation for 10 min at 15,000 rpm. The supernatant was then spectrophotometrically quantified at 350 nm to determine LUT content. Drug-free exosome-loaded MN arrays were used as blank. All measurements were conducted in triplicate.

To evaluate the homogeneity of drug distribution within the arrays, the MN arrays were cut into two equal halves by weight, dissolved using the same procedure, and analyzed for drug content [27].

#### Mechanical properties

A TA-XT2 Texture Analyzer (CT3, Brookfield, USA) operating in compression mode was used to assess the compression properties of MN arrays. The MN array was pressed against an aluminum block under a 30 N force and 0.1 mm/s compression rate. The heights of the MN arrays were measured both before and after applying the compression load with the aid of a stereomicroscope (C-B10 +, Optika microscope, Italy). The percentage reduction in MN height due to compression was calculated using the following formula: [27]

$$\%\, Height\, reduction= \frac{height\, before\, compression-height\, after\, compression }{height\, before\, compression}\times 100$$

#### Insertion depth

Parafilm M^®^ was employed as a scleral simulant following a slightly modified procedure described by Albadr et al. [26]. It was folded into a three-layered film, and the MN arrays were pressed against this parafilm laminate under 10 N force and 0.1 mm/s compression rate and held for about 30 s (to mimic manual insertion of MNs in the sclera) using TA-XT2 Texture Analyzer. This was followed by careful removal of the array from the parafilm. The holes created by the insertion in each layer were examined after unfolding the parafilm layers with the aid of a C-B10 + stereomicroscope (Optika microscope, Italy).

#### Ex vivo dissolution of MNs

MN arrays’ dissolution behavior was studied directly on excised bovine scleral tissue. The tissue was first laid flat. A dental wax board was used to first lay the tissue followed by the MN array insertion into the center of the scleral tissue. The MN was left inserted for specific durations (0, 30, 60, 90 s). At each interval, the arrays were removed and examined under a stereomicroscope (C-B10 +, Optika microscope, Italy). The remaining needle height was measured and recorded as a percentage of the original height over time [28].

### Ex vivo permeation study

Franz diffusion cells were utilized to perform the permeation study ex vivo of MN arrays [29]. The excised bovine scleral tissue was fixed on the donor chamber with the aid of cyanoacrylate adhesive after being soaked in PBS (pH 7.4) for two hours before the investigation. Seven mL of 25% PEG 400 in PBS (pH 7.4) were added to the receptor chamber, which was then kept at 37°C. MNs were then applied to the sclera and held in place using finger pressure for 30 s and left to incubate. In another set of experiments, a gel matrix containing the same MN’s polymeric composition and amount was placed on the sclera and its permeation through the sclera was compared to the dissolving MN. At designated sampling intervals (1, 3, and 6 h), 200 µL was withdrawn from the receptor chamber and replaced with an equivalent volume of 25% PEG 400 in PBS (pH 7.4) to preserve sink conditions. HPLC was then used to analyze the collected samples. The HPLC assay was conducted following a previously reported method by Liu Y et al., with a minor modification using an Agilent 1260 series system (USA) [30]. The analysis utilized a C18 reversed-phase column (4.6 × 150 mm; Tosoh, Tokyo, Japan) with a mobile phase consisting of a 60:40 ratio of methanol and 0.5% v/v phosphoric acid (H_3_PO_4_) in water. The assay operated at a flow rate of 1.2 mL/min. LUT was detected at a wavelength of 350 nm and its retention time was 4.3 min. LUT’s calibration curve was generated using concentrations ranging from 0.5 to 10 μg/mL. LUT was quantified and corrected in samples withdrawn from the recipient chamber using the same equation in Section "In vitro drug release".

### Irritation test: Chorioallantoic membrane assay using hen's eggs (HET-CAM test)

The chorioallantoic membrane (HET-CAM) method using hen’s eggs was utilized to assess the ocular tolerability of the formulated product, in accordance with standard protocols [29]. The polymeric gel matrix of the MN served as the test samples (LUT@gel, EX@gel, LUT-EX@gel). The positive and negative controls were 0.1 M NaOH and NaCl (0.9% w/v), respectively. After getting rid of the eggshell above the air cell, the inner membrane was carefully separated. The highly vascularized chorioallantoic membrane (CAM) was exposed and treated with 300 μL of the tested samples and controls following the removal of the inner membrane. Over five minutes after the samples were applied, any signs of irritation, such as bleeding, lysis, or coagulation, were seen and noted.

### In vivo glaucoma-induced rabbit model

#### Animals

Healthy male adult albino New Zealand rabbits weighing between 2–2.5 kg with no visible eye abnormalities were purchased from the animal facility at the Faculty of Agriculture, Alexandria University. All experiments were performed in compliance with the guidelines set by the Institutional Animal Care and Use Committee (AU-06–2023-11–11-1–206), Faculty of Pharmacy, Alexandria University.

#### IOP measurement

Fifty-four albino New Zealand rabbits were divided into nine groups, and each group included six rabbits. Induction of glaucoma was achieved by applying 1% cortisone eye drops topically twice a day for 21 days [15]. Schoetz tonometer (K-6100, Germany) was used to measure the IOP, with eyes anesthetized using 1–2 drops of benoxinate hydrochloride eye drops (Benox^®^, Eipico Co., Egypt) before each measurement. Baseline IOP was recorded for each glaucomatous eye before treatment. A single application of the selected formulation (Dose approximately equivalent to 40 µg LUT either in MN or applied gel matrix) was applied locally to the right eye of each rabbit, while the left eye, which received no treatment, served as the positive control. IOP measurements were taken at 3, 24, 48, 72, 96, 120, 144, and 168 h post-treatment. Table 1 shows the different 9 treatment groups used during the experiment.

**Table 1:** Different treatment modalities used to evaluate the in vivo efficacy of the prepared formulations, where treatment was applied on the right eye, while the left eye received no treatment, serving as a positive control

Group	Treatment on the right eye
Negative Control	Untreated healthy rabbit (no glaucoma induced)
Blank gel	Unloaded gel matrix (no Exosomes or LUT)
Blank MN	Unloaded MN (no Exosomes or LUT)
EX@gel	Gel matrix loaded only with Ex
EX@MN	MN loaded only with Ex
LUT@gel	Gel matrix loaded only free LUT solution
LUT@MN	MN loaded only with free LUT solution
LUT-EX@gel	Gel matrix loaded with LUT-EX
LUT-EX@MN	MN loaded with LUT-EX

Either MN or gel was applied once with a dose equivalent to 40 µg LUT

The percentage IOP reduction at all time points was calculated using the following formula: [31]

$$IOP\, reduction \%= \frac{IOP\, before\, treatment-IOP\, after\, treatment }{IOP\, before\, treatment}\times 100$$

AUC, C_max_ (highest % reduction in IOP), and T_max_ (time taken to achieve C_max_) for IOP reduction over time across different treatment modalities were also calculated. At the end of the experiment, all nine groups of rabbits were sacrificed. The eyeball of a group of animals was separated and put in 10% v/v formalin so that the eye could be examined histologically. In a different set, a 30-gauge needle was used to remove the aqueous humor by passing through the middle of the anterior chamber and above the corneoscleral junction. After being collected, the aqueous humor was kept at −80°C for further analysis.

#### Oxidative stress measurement

Oxidative stress was measured in the withdrawn aqueous humor by measuring the level of glutathione peroxidase (GPx) using an activity assay kit (E-BC-K096-S, Elabsience, USA). Quantification was carried out following the guidelines provided by the manufacturer.

#### Assessment of protein levels of TNF-alpha and IL-8 biomarkers

Biomarkers involved in the pathogenesis of glaucoma were quantitatively evaluated in the aqueous humor. TNF-alpha (E-EL-RB0011, Elabscience, USA) and IL-8 (#E-EL-RB1142, Elabscience, USA) ELISA kits were used. Following the instructions of the manufacturer, the quantification was done, and the results were presented as absolute values for the various measured protein levels (pmol/mL).

#### Quantitative real-time reverse transcriptase polymerase chain reaction (qRT-PCR)

The fold change in gene expression of myocilin (MYOC), nuclear factor erythroid 2-Related Factor 2 (NRF2), tissue inhibitor of metalloproteinases (TIMP), and IL-1β genes was assessed using qRT-PCR. Table 2 shows the sequence of primers (forward and reverse) used for PCR amplification. GAPDH was used as an internal housekeeping gene to standardize relative levels of transcription. The relative fold change in gene expression in the different treatment samples relative to negative control samples was determined using the comparative threshold cycle (2^-ΔΔCt^) technique.

**Table 2:** Sequence of forward and reverse prime of rabbit myocilin (MYOC), Nuclear factor erythroid 2-Related Factor 2 (NRF2), tissue inhibitor of metalloproteinases (TIMP), IL-1β and GAPDH genes

	Forward	Reverse
Rabbit MYOC	CATCCGCCAGGTCTTTGAGT	CGTGTAGCCACCCCAAGAAT
Rabbit NRF2	TTCACATTTTCTGCCTCCGC	GTTTGGGAATGTGGGCAACC
Rabbit TIMP	CTTCTCTCAACGTTCCGGCT	GGATGCACAGGCAAACACTG
Rabbit IL-1β	GCACAACAGATCGCTTTGGG	TTCTCCAGAGCCACAACGAC
Rabbit GAPDH	GGAGAAACCTGCCAAGTATG	AAGAATGGGAGTTGCTGTTG

Furthermore, correlation analysis between molecular biomarkers on both measured gene and protein levels against clinical outcomes, specifically intraocular pressure (IOP) reduction across different animals within the same treatment group was done to examine how the molecular expression patterns align with physiological responses. Pearson correlation coefficient (r), coefficient of determination (r^2^), and p-value were also calculated.

#### Histological examination

After being separated from eyeballs preserved in 10% w/v formalin, the cornea, retinal, and scleral tissues were processed and embedded in blocks of paraffin wax. Blocks were then sliced into 5-um-thick slices to prepare sections. Hematoxylin and eosin (H&E) was used to stain different sections for general analysis. Finally, slides were seen using a Canon digital camera and a polarized light microscope (Carl Zeiss, Köln, Germany).

### Statistical analysis

Experiments were conducted in triplicate. Mean ± SD was used to express all the results. GraphPad Prism (Version 8.0.2, San Diego, CA, USA) was used to conduct the unpaired Student's t-test or one-way analysis of variance (ANOVA) at a level of significance of p ≤ 0.05. Multiple comparison was performed when required.

## Results and discussion

### In vitro characterization of bovine colostrum milk-derived exosomes

Milk-derived exosomes have several benefits over exosomes derived from other sources when it comes to treating glaucoma. Milk is a plentiful and easily sustainable source of exosomes. Additionally, collecting large amounts for therapeutic uses is easier and simpler [32]. Moreover, milk-derived exosomes are extremely biocompatible and unlikely to trigger any immunological reaction because they are naturally obtained from a common food source. The development of non-invasive treatment approaches benefits from the stability and integrity of this type of exosomes. Moreover, exosomes derived from milk include bioactive compounds that have anti-inflammatory and neuroprotective qualities, among other potential therapeutic benefits [33]. Thus, milk-derived exosomes are a viable option for additional study and advancement in glaucoma treatment.

In our study, bovine colostrum milk-derived exosomes were successfully isolated using the precipitation method. There are several benefits to utilizing polyethylene glycol (PEG) in the precipitation process to separate colostrum-derived exosomes. PEG-based precipitation reliably produces a large number of exosomes. Additionally, PEG reagents are widely accessible and affordable, making this approach cost-effective as it does not require complex procedures or complicated equipment. It is also worth mentioning that PEG-based precipitation preserves the integrity and biological activity of exosomes, which makes them suitable for a variety of downstream uses [34].

**Fig. 1:**
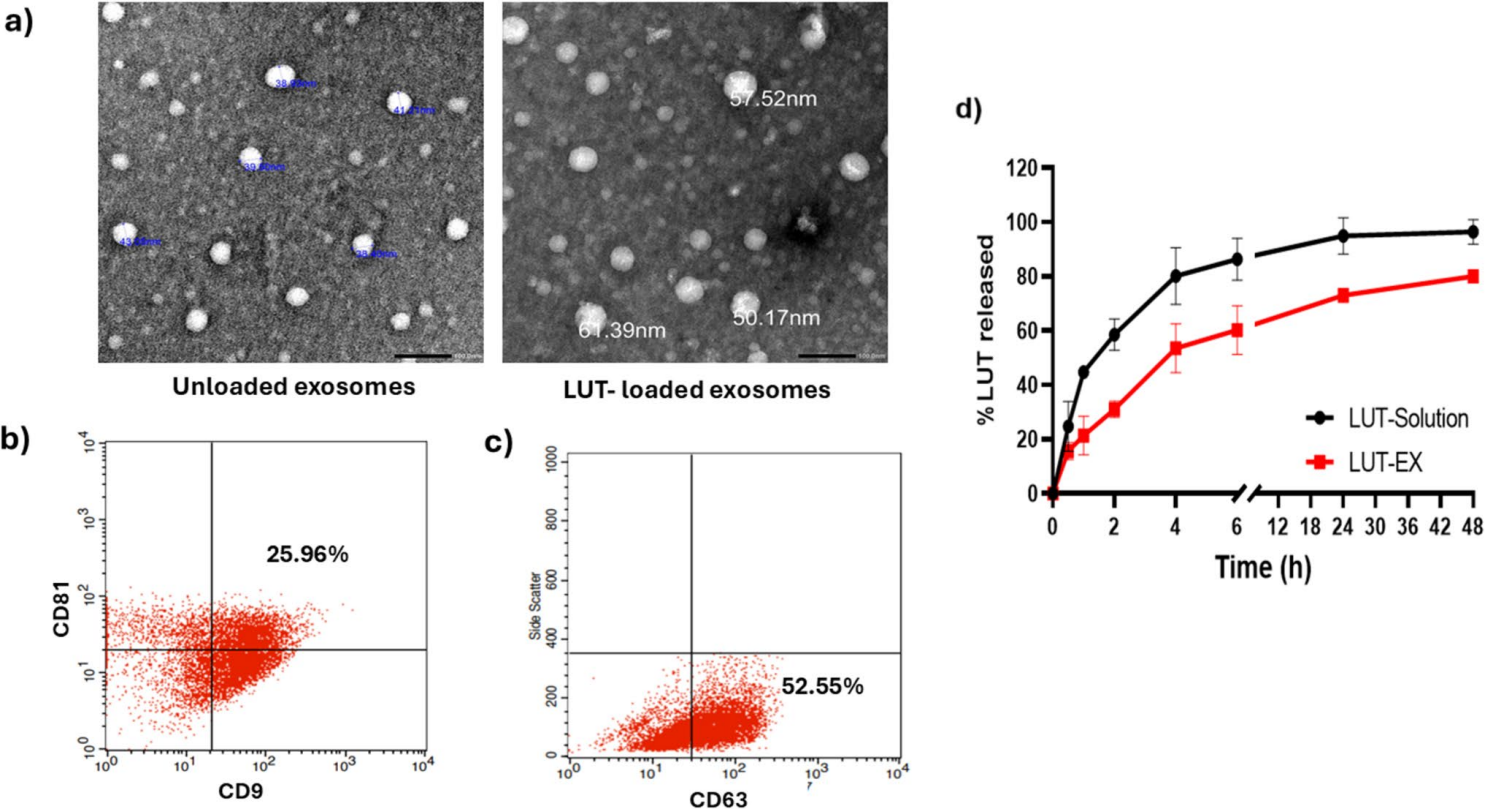
Characterization of the isolated colostrum milk-derived exosomes including **a)** Transmission electron microscope (TEM) of unloaded and loaded exosomes, **b)** Characterization of exosomes surface biomarkers (CD9 and CD81) determined by flowcytometry, **c)** Characterization of exosomes surface biomarker CD63 determined by flowcytometry, **d)** Profile of in vitro release of LUT free solution and LUT-loaded exosomes (LUT-EX) in 12mL 25% PEG (v/v) in PBS (pH 7.4) kept at 37 °C and 100 rpm to ensure sink conditions. Results are expressed as % LUT released, and the data presented is the mean of triplicate (mean ± SD)

The successful isolation of colostrum-derived exosomes was confirmed, as depicted in Fig. 1, which highlights their morphology, colloidal properties, and surface markers from bovine colostrum milk. TEM imaging verified the uniform spherical shape of these natural nanoparticles (Fig. 1a) both before and after drug loading, with average diameters of 45.18 ± 4.2 nm and 51.74 ± 2.9 nm, respectively. The slight increase in size suggests an insignificant change, indicating that the integrity of exosomes remained intact. The extracted exosomes’ average particle size measured by the Malvern zetasizer was 50.83 nm with a PDI of 0.13. This discrepancy may arise because TEM directly assesses the physical size of air-dried exosomes, while the zetasizer determines their hydrodynamic diameter, which includes the influence of the surrounding liquid layer causing the observed slight increase in particle size [35]. The results of Lei et al. align with our results, who reported a size of 83.2 nm for milk-derived exosomes [36] where both isolated exosomes showed a typical size of exosomes (<100 nm) when compared to other extracellular vesicles [37].

Particle concentration and zeta potential of the isolated colostrum milk-derived exosomes were assessed using the Malvern Zetasizer before. The analysis revealed a particle concentration of 1.54E+10 particles/mL and an average zeta potential of −21.89 mV, indicating that the extracted exosomes exhibit reduced susceptibility to excessive aggregation and precipitation; a desirable characteristic for an effective drug delivery system. These results align with earlier studies that reported a zeta potential of −17 mV for exosomes derived from bovine milk [38]. Additionally, previous studies by Lei et al. reported a yield of 7.24E+9 particles/mL for bovine milk-derived exosomes, which supports and aligns with our findings [36]. Both particle concentration and zeta potential were assessed for LUT-loaded EX to confirm exosomal membrane integrity after drug loading. Particle concentration for loaded EX was 1.52E+ 10 and zeta potential was around −16 mV, confirming that the exosomes kept their structure intact after drug loading.

Bradford protein assay was carried out to measure the content of protein within the isolated exosomes, where protein concentration directly reflected the exosome content [39]. The total protein content was 2.944 mg/mL, confirming the efficient isolation of exosomes using the applied method. This finding aligns with the results documented by González-Sarrías, et al. who reported that the protein content in the developed bovine milk exosomes was 1.49-2.61 mg/mL [40].

The lipid bilayer of exosomes contains specific membrane proteins, such as tetraspanins (e.g., CD81, CD9, and CD63), which are crucial for facilitating the fusion of exosomes with the membranes of recipient cells. Additionally, these proteins serve as molecular markers, aiding in the identification of exosomes across various experimental methods [32]. The milk-derived exosomes tested positive for surface markers CD81, CD9, and CD63, representing 29.77%, 39.99%, and 52.55%, respectively as illustrated in Fig. 1b and 1c. Based on the exosome characterization data, there is clear evidence that the extracellular vesicles we isolated qualify as exosomes in terms of their size, morphology, and surface marker expression, in accordance with the MISEV-2023 guidelines [41].

### Determination of entrapment efficiency of LUT

To optimize entrapment efficiency, two different concentrations of LUT solution (3 mg/mL and 5 mg/mL) were tested. The isolated exosomes exhibited entrapment efficiencies of 56.9% for the 3 mg/mL sample and 27.84% for the 5 mg/mL sample, suggesting an inverse relationship between concentration and loading saturation. This inverse relationship has been also observed by Gul et al. where entrapment of methotrexate decreased as its concentration increased upon loading in plasma-derived exosomes due to saturation and precipitation [42]. Similarly, Aqil et al. reported an entrapment efficiency of 53.9% for exosomes isolated from raw bovine milk [23]. Based on these results, the 3 mg/mL LUT concentration was selected for further use.

### In vitro release of LUT from LUT-EX

The release profile of LUT from LUT-EX was evaluated in vitro using 25% v/v PEG 400 in PBS (pH 7.4) at 37 °C, showing a biphasic release profile (Fig. 1d). LUT exhibited an initial burst release from LUT-EX within the first 1 h, releasing around 20% of LUT, followed by a sustained release over 48h. In comparison, free drug displayed nearly complete (100%) release within 24 h with an initial burst release of about 50% in the first hour. The burst release behavior of LUT-EX could be attributed to surface-adsorbed drug molecules, while the sustained release is due to the gradual diffusion of LUT encapsulated within the exosomes [43].

### In vitro studies of cell culture

#### In vitro cytotoxicity studies

**Fig. 2:**
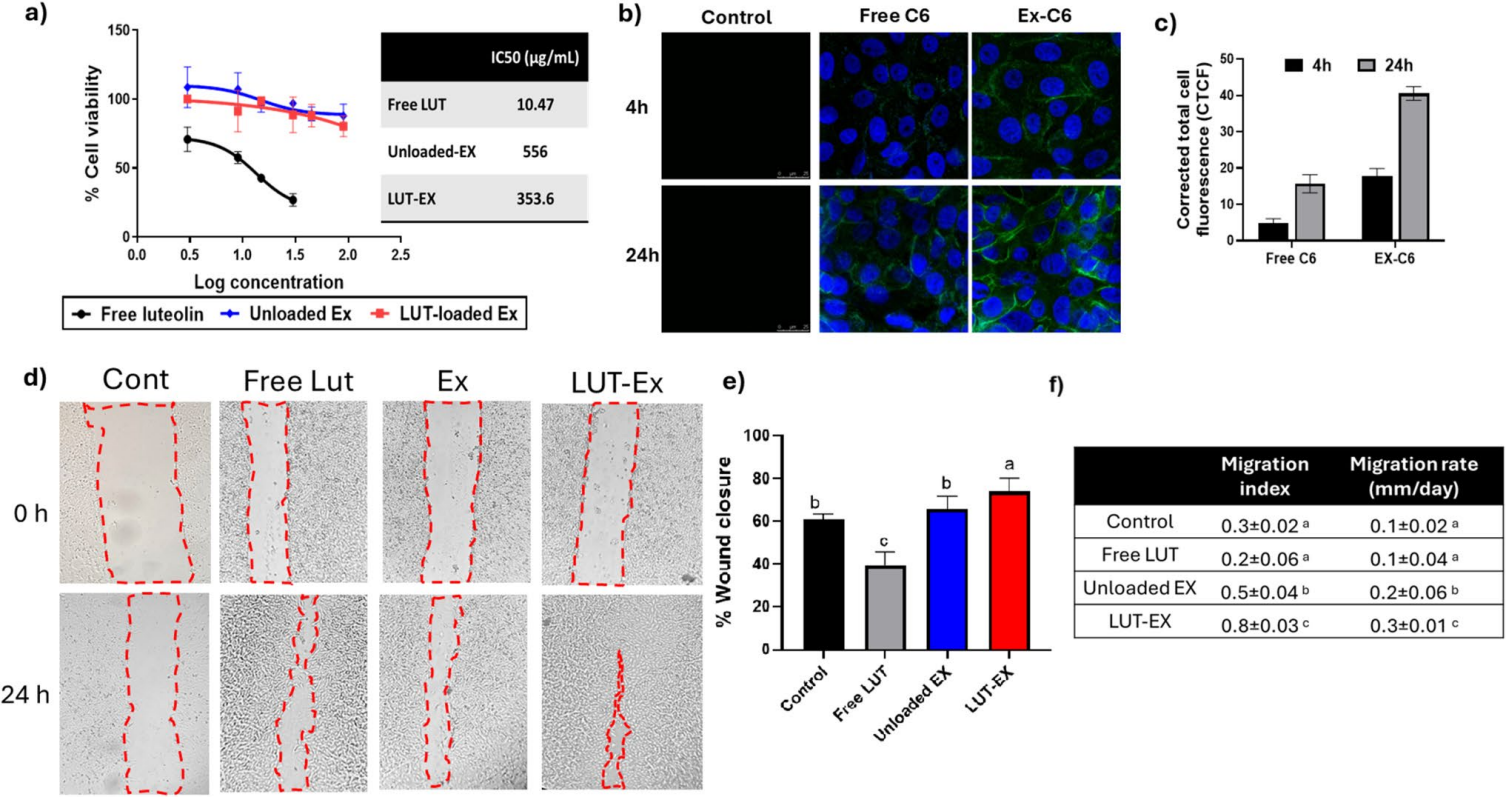
Evaluation of CSF: **a)** Cells’ viability with increasing concentrations of LUT, EX, and LUT-EX after 24 h, **b)** Images captured by confocal laser microscopy demonstrating the cellular association of C6 free solution and C6-EX 4 h and 24 h post incubation, **c)** Quantitative representation of the corrected calculated total fluorescence intensity. (n = 6, mean ± SD,) (p ≤ 0.001), **d)** Representative images for the migration assay: to different treatments’ migration activity (magnification × 20), and **e)** Calculated percentage closure of scratched area and **f)** calculated migration index and migration rate of cells after treatment with different formulations after 24h (n = 6, mean ± SD,). One-way ANOVA was utilized for data analysis, followed by Tukey’s post-hoc test to compare groups. Means of similar symbols were statistically insignificant: a > b > c (p ≤ 0.05)

CSF cells were used to assess the cytotoxicity of LUT, unloaded EX, and LUT-loaded exosomes on normal corneal tissues. As shown in Fig. 2a, LUT-EX showed higher cell viability compared to free LUT at all concentrations with IC_50_ values of 10.47, 556, and 356.3 µg/mL for free LUT, unloaded EX and LUT-EX, respectively.

This suggests that loading LUT into exosomes decreases LUT’s cell cytotoxicity. Exosomes are extremely biocompatible and generally safe since they are naturally produced from cells or natural resources and are made up of lipids, proteins, and nucleic acids. Thus, compared to synthesized nanoparticles, they are less prone to cause immunological reactions [44]. The increased cell viability observed with LUT-EX relative to the free drug is in agreement with other studies that showed that drug-loaded exosomes have promising safety profiles in preclinical and clinical studies and are capable of delivering therapeutic payloads such as drugs, siRNA, and proteins with minimal toxicity [45].

#### Cellular association

The cellular association experiment was assessed to evaluate the effectiveness of the prepared exosomal delivery systems and their capability to reach and be absorbed by target cells in the eye. As mentioned earlier, exosomes are considered potential natural delivery systems for drugs, DNA, and other therapeutic agents. They are promising vehicles for targeted therapy because of their capability to enhance cellular uptake [46]. As shown in Fig. 2b and 2c, Ex-C6 showed significantly higher cellular uptake compared to free C6 at both time points (4 h and 24 h). Cellular uptake was more prominent after 24 h where the uptake of Ex-C6 increased by 3.3-fold compared to a 2.1-fold increase after 4 h. This indicates that EX-C6 is more effective in delivering the compound into cells, resulting in higher fluorescence. This is supported by previous studies showing that exosomes can improve the delivery of therapeutic agents [47]. Exosomes can target particular cells with precision due to their surface proteins and ligands. This targeting capacity helps minimize off-target effects by delivering therapeutic chemicals directly to the diseased cells [48].

Exosomes are known to facilitate the uptake of C6 into cells through clathrin-mediated endocytosis and micropinocytosis, a process where cells engulf the exosomes along with their cargo [49]. Moreover, the exosomal delivery system ensures that C6 is retained within the cells for a longer duration, reflecting the enhancement in the drug’s therapeutic efficacy. The lipid bilayer membrane of the exosomes, which is similar to that of the cell membrane also enables them to fuse with target cells and deliver their cargo directly into the cytoplasm, bypassing endocytic pathways [50].

#### Cellular proliferation and migration evaluation using scratch assay

In glaucoma research, wound healing assays on corneal fibroblasts are crucial for evaluating the therapeutic potential of drugs such as LUT that target the damage in the trabecular meshwork (TM) or other ocular tissues for glaucoma treatment. These experiments aid in assessing how drugs affect cellular processes that are essential for preserving IOP and aqueous humor outflow, such as migration, proliferation, and extracellular matrix (ECM) remodeling.

In this study, tissue regeneration and cell migration capabilities were assessed via a scratch model assay. The % wound closure, migration index and migration rate were calculated which are crucial for understanding cell motility and repair mechanisms in the TM and RCGs. The migration index helps assess the efficiency of cell movement, indicating how well cells contribute to wound healing and tissue regeneration. On the other hand, the migration rate quantifies the speed of cell migration, providing insights into how quickly damaged tissues may recover [51]. As shown in Fig. 2d-f, LUT-EX showed a significantly higher (p ≤ 0.05) percentage of cell migration after 24 h (75% closure in the scratched area with migration index of 0.8 ± 0.03 and migration rate of 0.3 ± 0.01 mm/day) when compared to the untreated cells, followed by unloaded Ex (65%) and Free LUT (40%). This suggested that LUT-EX significantly enhanced cell migration compared to other treatments. The significant cell migration and proliferation observed with LUT-EX treatment suggest that exosome-mediated delivery of LUT can enhance the cell proliferation and extracellular matrix (ECM) remodeling process. This could be due to the anti-inflammatory and antioxidant properties of LUT, which are more effectively delivered by exosomes. It is worth mentioning that exosomes can promote both the migration and proliferation of fibroblasts and keratinocytes, which are crucial for ECM remodeling [52]. The presence of LUT within exosomes further enhances these processes, leading to faster wound closure.

### Fabrication and characterization of MNs

#### Fabrication and morphological examination

Microneedles were developed using a polymeric combination of PVP and PVA to ensure mechanical strength and structural stability during storage and flexibility, which is crucial for maintaining structural integrity during skin insertion [53]. Both PVP and PVA are biocompatible and biodegradable, consequently, they are safer for biomedical applications compared to MNs made from non-biodegradable materials like metals. PVP/PVA MNs can be designed to control the release rate of drugs, allowing for sustained and targeted delivery, which is a significant advantage over some other MN types [54]. The combination of these polymers enhances the penetration capabilities of MNs, ensuring the effective delivery of therapeutic agents [53]. In comparison, MNs made from other materials, such as metals or ceramics, may offer higher mechanical strength but lack the biodegradability and biocompatibility of PVP/PVA MNs. Additionally, MNs made from materials like silicon or glass can be more challenging to fabricate and may not provide the same level of controlled drug release [55].

**Fig. 3:**
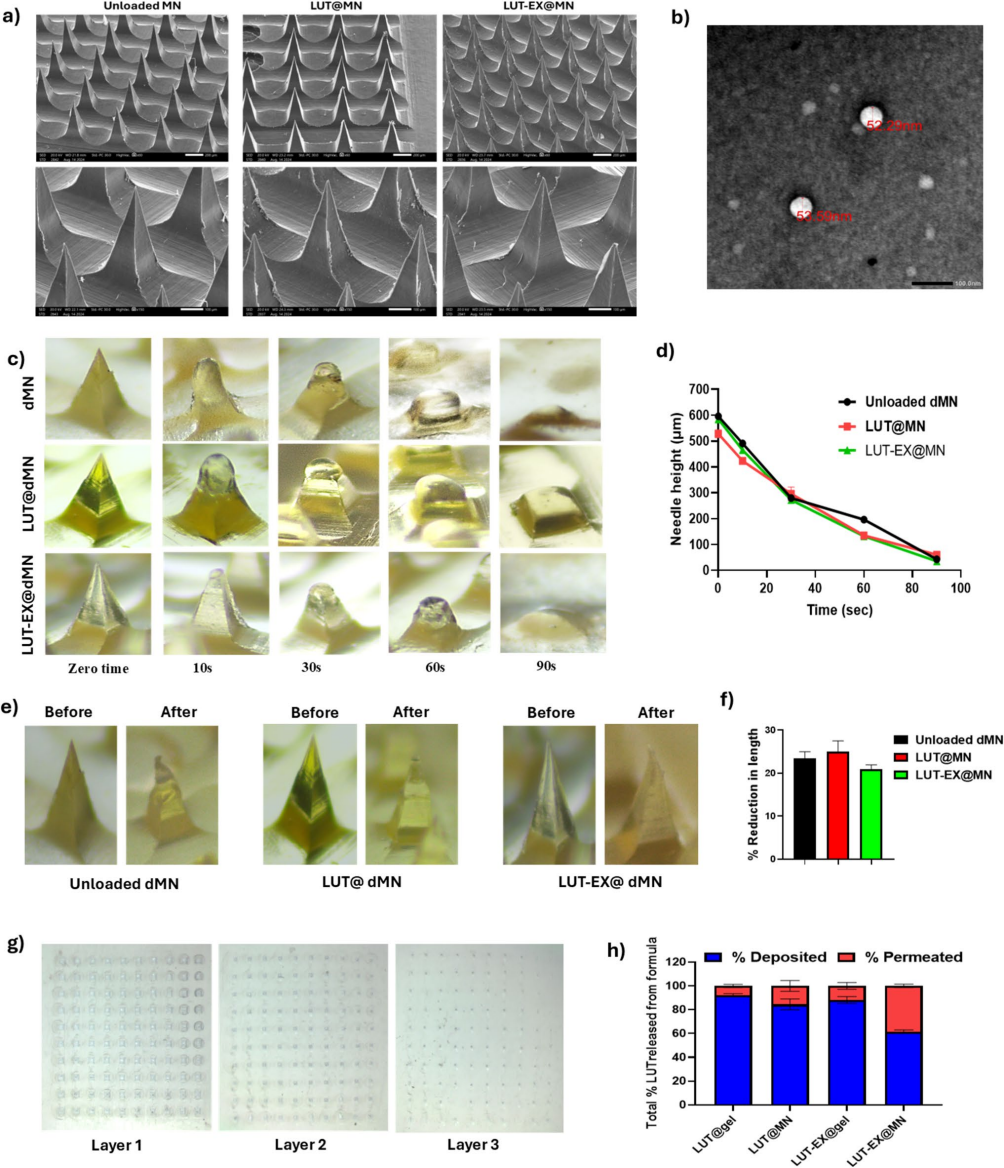
Characterization of prepared MNs: **a)** SEM image for morphological examination of unloaded MN, LUT@MN and LUT-EX@MN at magnification × 60 and × 250, scale bar represents 60 μm and 250 μm, **b)** TEM images demonstrating the preservation of vesicular nature of exosomes after incorporation in MNs, **c)** Images captured using a stereomicroscope at various times after unloaded MN, LUT@MN, and LUT-EX@MN were inserted into freshly dissected bovine sclera demonstrating their dissolution. Magnification 4.5×, **d)** Percentage of the length of needle remaining post dissolution at various time intervals for either blank dMN, LUT@MN, or LUT-EX@MN (mean ± SD, n = 3), **e)** Images showing MN height of unloaded MN, LUT@MN, and LUT-EX@MN before and after compression using the texture analyzer. (mean ± SD, n = 5), **f)** Graphical representation of the % reduction in length after compression, **g**) Using Parafilm M^®^ at a magnification of 4.5x, micrographs of stereomicroscope illustrate the in vitro insertion behavior of MN and display the microneedle array after insertion in three distinct Parafilm layers. and **h)** A bar graph displaying the percentage of LUT that either penetrated or was deposited in freshly dissected bovine scleral tissue following six hours of exposure to various treatments. (mean ± SD, n = 3)

Propolis was incorporated into the polymer blend due to its demonstrated effectiveness in treating ocular diseases, likely due to its antiglaucoma, antioxidant, anti-inflammatory, and neuroprotective properties [56]. The MN arrays (10 × 10 needle arrangement) had a height of 580.72 ± 10.58 μm and contained 100 microneedles. Water evaporation while drying was shown to cause an average 3.3% decrease in height when compared to the actual height of silicone mold (600 μm) [57]. The MN array's appearance and the pyramidal structure of unloaded, LUT-loaded, and LUT-EX-loaded MN are shown in Fig. 3a. It has been reported that pyramidal microneedles were more forceful than conical ones because, at the same base diameter or width, they possess larger cross-sectional area, which makes it easier to introduce into the retina and reduces the risk of bending or breaking during the insertion procedure [5]. The drug content in MN was measured using spectrophotometrically, showing that LUT@MN or LUT-EX@MN array contained approximately 39.7 ± 1.27 μg of LUT. To verify uniform drug distribution, the MN patches were divided into equal halves, and the LUT content in each half was assessed. The analysis confirmed even drug distribution, where each half contained approximately equal amounts with an average of 19.59 ± 1.09 μg of LUT in each half.

To ensure the integrity of LUT-EX following their incorporation into the MN matrix, deionized water was used to dissolve exosome-loaded MNs, where aliquots were analyzed using TEM, which confirmed the preservation of their vesicular structure (Fig. 3b).

#### Ex vivo dissolution test

To determine the ideal application time for dissolving MNs on scleral tissue, a dissolution study was performed. Figure 3c presents the dissolution times for both blank and exosome-loaded MNs inserted into the sclera. The results indicated that the MNs, whether blank or loaded with exosomes, took over 1 min to fully dissolve (Fig. 3d). This characteristic makes them well-suited for intrascleral drug delivery, as rapid dissolution (less than 1 min) could cause the sharp tips to dissolve before fully penetrating the scleral tissue, resulting in drug deposition on the surface as opposed to inside the sclera thus lowering bioavailability [58].

#### Mechanical properties

For effective ocular drug delivery, MNs must be strong and sharp in order to effectively penetrate the scleral tissue and endure compression without breaking. Evaluating the mechanical strength of the MN array is thus critical. A predefined force of 30 N was selected as it surpasses the 10 N force necessary for effective implantation into ocular tissue [59]. Figure 3e depicts the percentage of height reduction in MNs when subjected to a 30 N force, demonstrating their ability to withstand sufficient pressure for successful tissue implantation. The MNs displayed adequate mechanical strength, with a height reduction of less than 25% under compression (Fig. 3f). Furthermore, there was no significant difference (p > 0.05) between blank MNs and those loaded with exosomes, implying that the inclusion of drug-loaded exosomes in the MNs did not compromise their durability.

#### Insertion depth

To evaluate the efficiency of trans-scleral insertion, Parafilm M^®^; an artificial folded membrane designed to mimic the layers of the sclera was used to test the insertion capability of the prepared MNs [26]. The parafilm was folded to get the three layers with a total thickness of 0.39 mm since the thickness of the parafilm sheet is 0.13 mm, and that of the human scleral tissues is around 0.67 mm [60]. This is to represent the penetration into the mid-scleral region rather than total scleral region without piercing through. The insertion test was conducted using a texture analyzer, previously employed for compression testing in compression mode, to measure the MN’s capability to insert into the layers of Parafilm, as depicted in Fig. 3g. Images in Fig. 3g illustrate complete penetration through three Parafilm layers. According to this, the therapeutic payload can be successfully distributed into the deeper layers of the human sclera once MNs have been inserted and the polymer matrix has biodegraded, thereby avoiding the scleral barrier. Additionally, the MNs penetrate only the mid-layer of the sclera, where they become soft and dissolve, rather than passing through the entire sclera. This is important to prevent getting in contact with or damaging highly sensitive ocular tissues like the retina and the choroid and enable delivery of the drug in a painless and minimally invasive way. Therefore, the insertion depth of the exosome-loaded MNs offers an optimal balance between delivery efficiency and patient comfort, making them ideal for effective transscleral drug administration [29].

#### Ex vivo permeation study

The impact of MNs on the permeation of LUT across the sclera was evaluated using the Franz-diffusion cells set-up. Fig. 3h illustrates the percentages of LUT that permeated and deposited when delivered either freely or encapsulated in colostrum-derived exosomes, using a gel (MN gel polymer matrix) or MNs. The percentage of LUT permeated following the application of LUT@MN was approximately two times higher than that achieved with the gel formulation. Furthermore, using LUT-EX@MN resulted in 40% of the loaded LUT permeating the sclera within 6 h, compared to just 15% from LUT-EX@gel, representing a ~2.6-fold increase in permeation. This significantly achieved enhancement in the permeation of LUT from MNs in comparison to the gel matrix highlighting their promising ability in improving drug delivery through the sclera.

### Ocular tolerance test

**Fig. 4:**
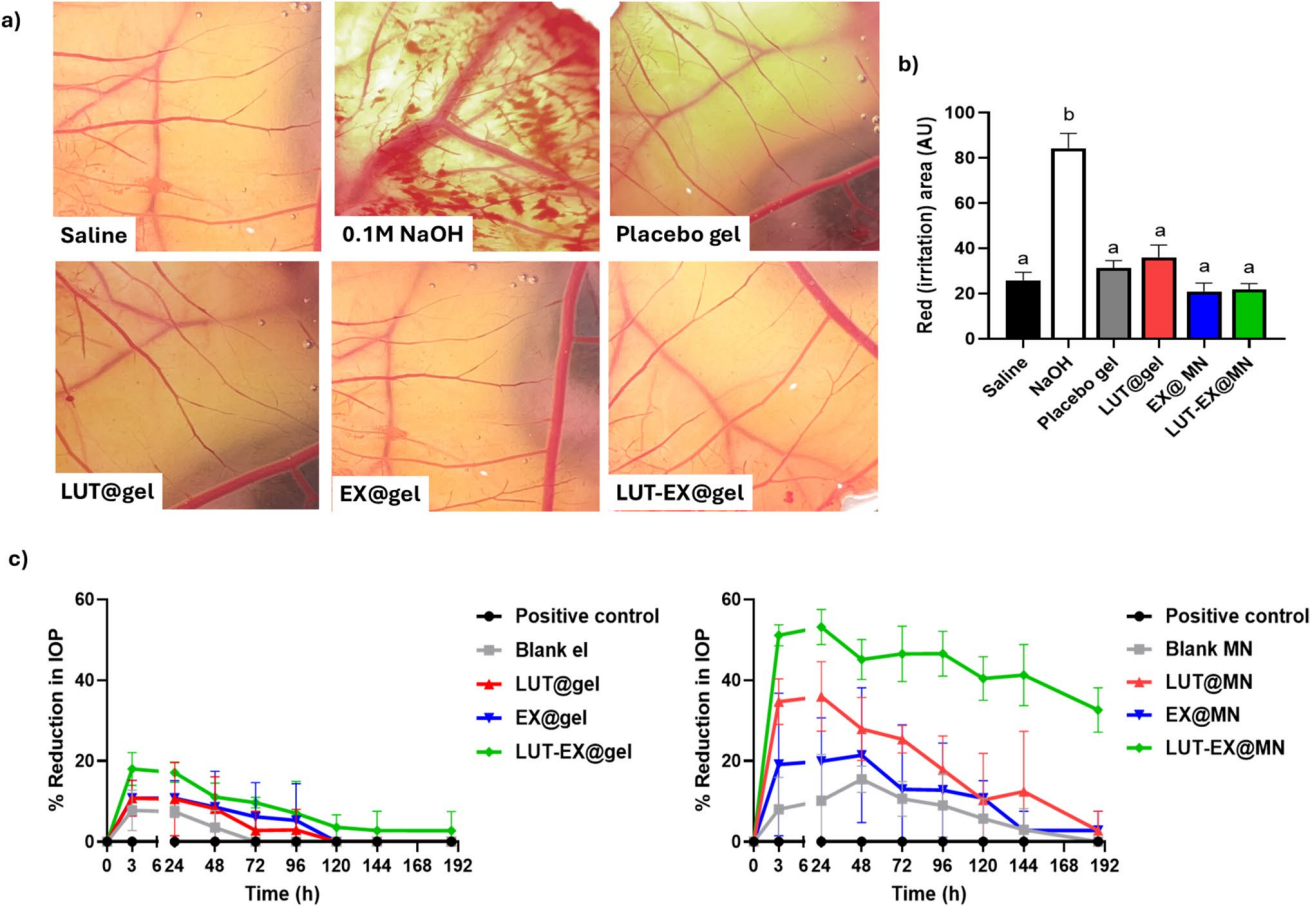
**a)** Images of chorioallantoic membrane test conducted on hen’s eggs (HET-CAM) following different treatments at room temperature for prediction of the potential of ocular discomfort and irritation potential. **b) **Scoring of ocular irritation in each sample via measuring the red irritated area and **c)** Percentage reduction in intraocular pressure following topical ocular insertion of a single dosage of different fabricated MN and their corresponding gel matrix over a period of seven days following glaucoma induction in rabbits

The eye is a highly sensitive and delicate organ, vulnerable to foreign substances or chemicals. To ensure safety, the prepared MN matrix was tested using the HET-CAM method; a fast, sensitive, and cost-effective test. As a model positioned between the systems of in vivo and in vitro studies, it circumvents ethical and legal concerns. The CAM of the chick embryo, consisting of veins, arteries, and capillaries, exhibits an inflammatory response to injury similar to that of rabbit conjunctival tissue. As illustrated in Fig. 4a and b, the formulations were evaluated against a negative control (normal saline, considered non-irritant) and a positive control (0.1 M NaOH, considered irritant). The scores were documented following the scoring system outlined by Gupta et al. According to this scoring system, all of the developed formulations exhibited a zero score for confirming their non-irritant properties [61].

### In vivo evaluation of the efficacy of LUT-EX@MN in the glaucoma-induced rabbit model

The anti-glaucoma potential of LUT-EX@MN was evaluated against various control groups (negative control, positive control, blank MN) and the same groups in the polymeric matrix before casting them into MN (blank gel, EX@MN, EX@gel, LUT@MN, LUT@gel, and LUT-EX@gel). These different formulations were tested in rabbits with glaucomatous right eyes over one week following a single application of the tested formulations. As depicted in Fig. 4c, gel-based formulations did not show a significant decrease in IOP through one week (p > 0.05), likely due to their limited pre-corneal retention time caused by blinking and nasolacrimal drainage [62]. However, LUT-EX@gel significantly lowered IOP within the first three hours (18% ± 3.3%), though this effect was not sustained over seven days as observed from the decrease in percent reduction of IOP (Fig. 4c). In contrast, LUT@gel did not show a comparable reduction in IOP during the same period (10.7% ± 3.5%). This difference may be attributed to the ability of exosomes to efficiently deliver their cargo to ocular tissues. Noticeably, EX@MN lowered IOP over a period of 48 h, highlighting the IOP-reducing properties of milk-derived exosomes (Fig. 4c). This observation is in agreement with previous findings by Seong et al., who reported that exosomes could help manage glaucoma by lowering IOP [63]. Of all the formulations tested, LUT-EX@MN demonstrated the most pronounced reduction in IOP, achieving approximately a 50% decrease within three hours, which was maintained over seven days (Fig. 4c). It is important to note that LUT-EX@gel did not achieve the same effectiveness as LUT-EX@MN, emphasizing the role of MNs in improving LUT-EX permeability. This observation is in line with the ex vivo permeation results. Moreover, this finding supports the capability of dissolving MNs to ensure sustained drug release [55]. The area under the curve (AUC) for IOP reduction across different formulations has been also calculated as shown in Table 3. The use of AUC for IOP reduction is well-established in glaucoma research, as it provides a more comprehensive assessment of treatment efficacy by accounting for both magnitude and duration of pressure control. Unlike single time-point measurements, AUC reflects real-world IOP fluctuations, making it a critical metric in evaluating sustained therapy. AUC is directly correlated with the extent and duration of the therapeutic effect. [64]. As shown in Table 3, AUC, C_max_ (highest % reduction in IOP), and Tmax (time taken to achieve C_max_) were calculated. Gels in general exhibited smaller AUC, lower C_max,_ and shorter T_max_ when compared to their corresponding MN formulations indicating the effect of MN in bypassing the corneal barrier and delivering the drug directly into the deeper ocular tissues. All gels exhibited shorter Tmax compared to MN (3h vs 24 or 48 h in MN) indicating rapid effect. Within gel formulations, there was no significant difference between blank@gel, LUT@gel, and EX@gel with regard to their AUC, C_max_ and T_max_. However, LUT-EX@gel showed approximately 1.8-fold increase in AUC reaching a C_max_ of 18 ± 4.1% after 3 h. The selected microneedles formulation (LUT-EX@MN) exhibited the highest AUC value among all formulations with 5.3-fold increase in AUC compared to LUT-EX@gel. The increase in C_max_ (53.2 ± 4.4%) and delayed T_max_ (24h) reflects the potential role of MN or exosome-based systems in sustaining drug release and improving therapeutic efficacy for ocular conditions like glaucoma highlighting the synergistic benefits of trans-scleral delivery and bioactive carrier systems. These findings strongly support the potential of biologically inspired and minimally invasive platforms for effective ocular drug delivery.

**Table 3:** AUC, C_max_ (highest % reduction in IOP) and T_max_ (Time taken to achieve C_max_) for IOP reduction over time across different treatment modalities (mean ± SD, n = 6)

	Blank GEL	LUT@GEL	EX@GEL	LUT-EX@GEL
AUC	312.2 ± 107.6 ^a^	632.9 ± 56.5 ^a^	818.9 ± 105.9 ^a^	1542.8 ± 97.3 ^b^
C_max_ (%)	7.8 ± 1.4	10.8 ± 4.4	10.8 ± 3.4	18.1 ± 4.1
T_max_	3	3	3	3
	Blank MN	LUT@MN	EX@MN	LUT-EX@MN
AUC	1407.7 ± 155.7 ^a^	3997.6 ± 634.3 ^d^	3012.5 ± 324.1 ^c^	8219.4 ± 110.1 ^e^
C_max_ (%)	15.4 ± 3.2	35.9 ± 8.6	21.4 ± 6.7	53.2 ± 4.4
T_max_ (h)	48	24	48	24

One-way ANOVA was used for data analysis which was followed by Tukey’s post-hoc test for group comparisons. Means of similar symbols were statistically insignificant: a < b < c < d < e (p ≤ 0.05)

### Assessment of gene expression and protein levels of various biomarkers participating in the management of glaucoma

**Fig. 5:**
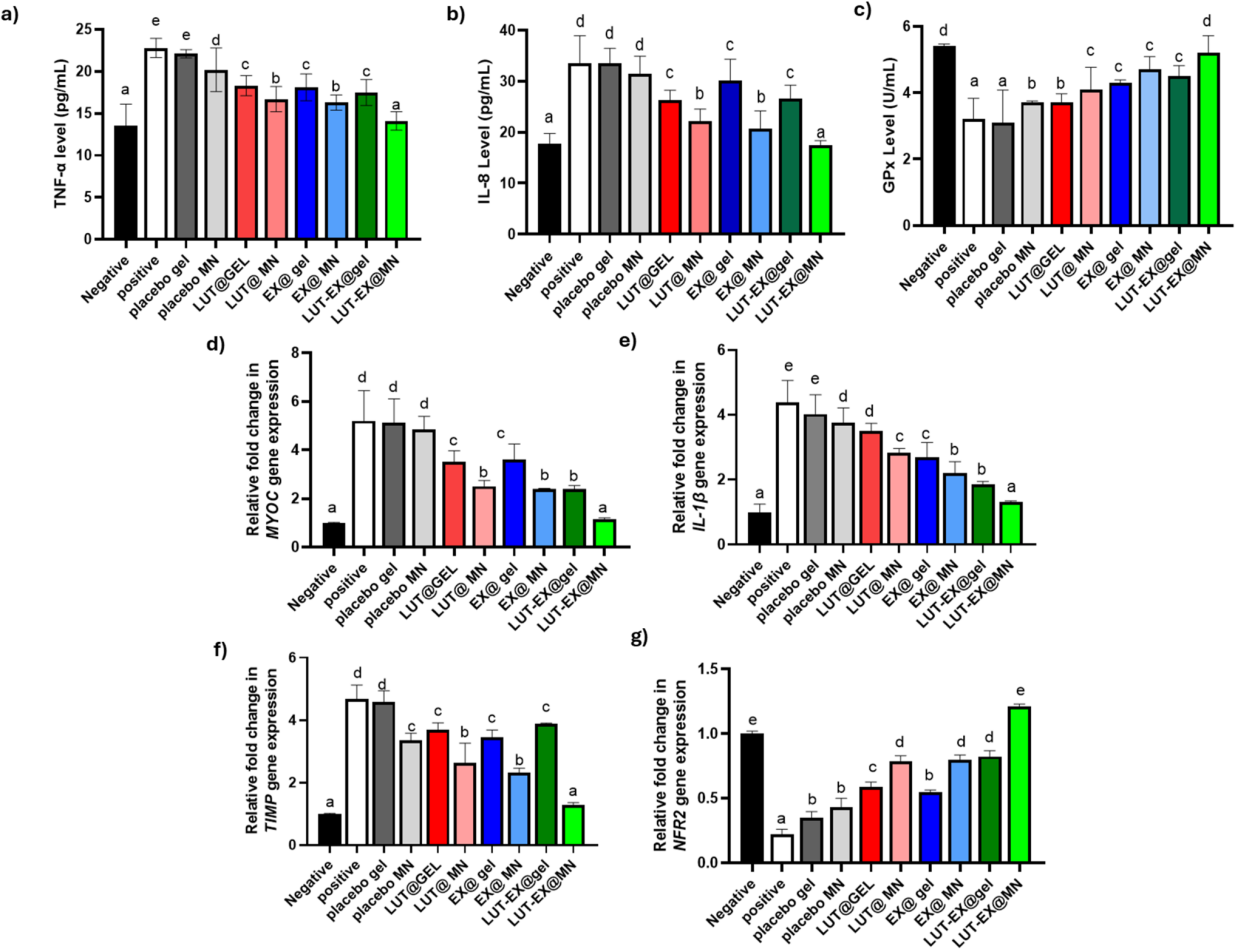
Analysis of glaucoma biomarkers in aqueous humor of different treated modalities. Protein levels of **a**) TNF-α, **b**) IL-8, and **c**) activity of GPx were measured quantitatively by ELISA. Fold change in gene expression levels of **d**) MYOC, **e**) IL-1β, **f**) TIMP, and **g**) NRF2 measured and normalized to the expression of GAPDH which acts as the housekeeping gene by qRT-PCR using (2^-ΔΔCt^). (mean ± SD, n = 3). One-way ANOVA was used for data analysis which was followed by Tukey’s post-hoc test for group comparisons. Means of similar symbols were statistically insignificant a < b < c < d < e and all of the p-values are ≤ 0.05

RGCs exposed to stress caused by IOP elevation undergo apoptosis, leading to progressive atrophy. Oxidative stress and cytokine release further contribute to the deterioration of glaucoma. In glaucomatous eyes, studies have reported increased TNF-α protein levels, elevated TNF-α gene expression in glial cells, and upregulated TNF-α receptor 1 in RGCs [65]. Figure 5a shows that the positive control group receiving no treatment exhibited significantly higher (p ≤ 0.05) TNF-α protein levels (22.8 ± 1.1 pg/mL) in comparison to the negative healthy control group (13.5 ± 2.6 pg/mL). Remarkably, TNF-α levels achieved with LUT-EX@MN treatment (14.1 ± 1.1 pg/mL) were statistically insignificant compared to those of the negative control group. Evidence from research supports this conclusion, suggesting that LUT may be a promising treatment for reducing inflammation and suppressing pro-inflammatory cytokines. Supporting these findings, previous studies done both in vitro and in vivo have shown that LUT modulates multiple signaling pathways associated with inflammation and effectively suppresses key inflammatory mediators, particularly TNF-α [66].

Furthermore, several studies examining cytokine concentrations in aqueous humor samples have identified elevated levels of cytokines, such as IL-8 [67]. As illustrated in Fig. 5b, the untreated positive control group showed significantly increased (p ≤ 0.05) IL-8 expression (33.5 ± 5.5 pg/mL) compared to the healthy negative control group (17.8 ± 1.96 pg/mL). Importantly, IL-8 levels following treatment with LUT-EX@MN (17.5 ± 0.9 pg/mL) were close to those of the negative control group. In an attempt to investigate the anti-inflammatory effect of LUT, Hytti et al. examined the effects of LUT on ARPE-19; the human retinal pigment epithelial cell line, assessing its potential to reduce intracellular inflammation. In addition, it has been documented that treatment with LUT led to a decrease in IL-8 levels and suppression of the inflammatory response in stressed ARPE-19 cells [68].

On the other hand, glutathione peroxidase (GPx) is a vital antioxidant enzyme that plays a role in neutralizing reactive oxygen species (ROS). In glaucoma patients, the heightened need for antioxidants like GPx results in their depletion [69]. As depicted in Fig. 5c, the untreated positive control group displayed significantly (p ≤ 0.05) reduced GPx activity (3.2 ± 0.6 U/mL) when compared to the healthy negative control group (5.4 ± 0.1 U/mL). Expression of GPx following treatment with LUT-EX@MN has increased significantly and its levels (5.2 ± 0.5 U/mL) were comparable to those in the negative control group. In line with these findings, Yuan et al. investigated the antioxidant properties of LUT by examining glutathione and glutamate levels in rabbits with glaucoma. The results showed that LUT treatment preserved endogenous aqueous humor glutathione levels, suggesting that LUT is not only effective in reducing IOP but also offers protection against oxidative damage and the advancement of glaucomatous neurodegeneration [15].

The MYOC gene encodes myocilin, a protein predominantly located in the trabecular meshwork (TM) near the cornea of the eye. Overexpression, misfolding, and aggregation of MYOC proteins induce endoplasmic reticulum (ER) stress and initiate the unfolded protein response (UPR) via the ER's homeostatic mechanisms. When the UPR fails to clear misfolded proteins through proteasomal degradation, cells are unable to resolve ER stress, leading to apoptosis and subsequent degradation of TM tissue. This ER stress response and resulting cell toxicity caused by misfolded MYOC are widely recognized as a key mechanism in the pathogenesis of glaucoma [70]. At the gene expression level, Fig. 5d illustrates that the positive control group exhibited a significantly higher fivefold increase in MYOC gene expression when compared to the negative control group. However, treatment with LUT-EX@MN effectively reversed this increase, resulting in the lowest MYOC gene expression, which was a 1.16-fold increase compared to the levels observed in the negative control group. It is noteworthy to mention that, extracellular vesicles and exosomes from diverse sources have been investigated as potential therapeutic options for glaucoma-related damage [71]. Supporting this, Pan et al. demonstrated that exosomes originating from umbilical cord mesenchymal stem cells enhanced RGC survival in a mouse model [72]. Consistent with these findings, our results suggest that colostrum-derived exosomes may represent a promising strategy for glaucoma therapy.

Moreover, it has been documented that NRF2 also plays a critical role in mediating the antioxidant response, safeguarding RGCs and glial cells from damage induced by elevated IOP. Reduced NRF2 levels weaken this antioxidant defense, resulting in increased oxidative stress and accelerated degeneration of RGCs [73]. As illustrated in Fig. 5g, the LUT-EX@dMN group demonstrated the highest NRF2 gene expression and showed a 1.21-fold increase in comparison to the negative control group, highlighting its potential to enhance cellular antioxidant defenses. Similarly, Gong et al. demonstrated that chlorogenic acid (CGA) treatment elevated NRF2 protein levels in retinal tissues, suggesting that CGA offers protective effects against glaucoma and holds promise as a potential therapeutic agent for the disease [74].

To further assess the impact of LUT-EX@MN in glaucoma, we analyzed the expression levels of tissue inhibitor of metalloproteinases-1 (TIMP1). A group of proteolytic enzymes called matrix metalloproteinases (MMPs) is responsible for breaking down extracellular matrix (ECM) components. They are essential for many biological functions, such as development and tissue remodeling in both healthy and pathological conditions. MMP and TIMP expression levels control the activity of these enzymes. IOP homeostasis is maintained in the eye by MMP-mediated ECM turnover within the TM, which lowers outflow resistance in the traditional outflow channel [75]. In glaucoma, alterations in the balance between MMP and TIMP as well as the reduced MMP activity in the aqueous humor have been observed, contributing to abnormal ECM buildup, increased aqueous humor outflow resistance, and elevated IOP [76]. Our results demonstrated that the developed LUT-EX@MN led to a significant decrease in TIMP1 levels by only 1.28-fold change compared to the negative control group, thereby contributing to its IOP-lowering effect (Fig. 5f). These findings are supported by a study on human cultured non-pigmented ciliary epithelial cells that examined the effects of prostaglandin analogs (PGAs); latanoprost, bimatoprost, and tafluprost which are commonly used to lower IOP in glaucoma patients. The study measured mRNA levels for MMPs and TIMPs after exposure to the free acid forms of these PGAs, revealing a dose-dependent reduction in TIMP mRNA levels [76].

IL-1β, however, plays an essential role in mediating cell death and inflammation, both of which are crucial factors in glaucoma progression. Increased levels of IL-1β have been detected in the aqueous humor of glaucoma patients, indicating its involvement in the disease's pathophysiology [77]. Our study found that LUT-EX@MN effectively reduced IL-1β levels by 1.32-fold, contributing to the antiglaucoma effect of the developed formulation (Fig. 5e). Similarly, Zhu et al. recently reported that LUT possesses anti-inflammatory properties by inhibiting IL-1β [78].

Interestingly, LUT-EX@MN treatment resulted in a significant alteration in TNF-α, IL-8, GPx, MYOC, NRF2, TIMP1, and IL-1β levels to closely resemble those of the healthy control group, outperforming the LUT-EX@gel therapy. Specifically, TNF-α and IL-8 levels dropped to 14.1 pg/mL and 17.5 pg/mL, respectively, in the LUT-EX@MN group. In contrast, the LUT-EX@gel group showed less significant reductions, with TNF-α at 17.5 pg/mL and IL-8 at 26.6 pg/mL. Additionally, the GPx level in the LUT-EX@MN group reached 5.2 U/mL, significantly higher than the 4.5 U/mL observed in the gel-treated group.

Furthermore, LUT-EX@gel exhibited 2.39, 0.824, 3.88, and 1.855-fold in the expression of MYOC, NRF2, TIMP1, and IL-1β genes, respectively. In comparison, LUT-EX@MN treatment led to 1.16-, 1.21-, 1.28-, and 1.32-fold changes in the expression of these same genes relative to the negative control group.

These results further support the superior efficiency of MNs over gels for LUT-EX delivery, as MNs can bypass the eye’s natural barriers and facilitate direct drug transport to targeted ocular tissues. Unlike gels, which rely on diffusion across the cornea and conjunctiva—often resulting in low bioavailability and the need for frequent administration, MNs create microchannels that enhance drug permeability, ensuring optimal therapeutic outcomes [62].

The correlation analysis between molecular biomarkers—spanning both gene and protein levels—and clinical outcomes, specifically intraocular pressure (IOP) reduction across different animals within the same treatment group was done to examine how the molecular expression patterns align with physiological responses. This could help in confirming the mechanism of action. As shown in Table 4 and Fig S1, a significant inverse correlation between protein markers (e.g., IL-6, TNF-α) and change in IOP values over time (represented as AUC) was observed. The Pearson correlation coefficient (r) was −0.94 and −0.96 for TNF- α and IL-6, respectively, while the correlation was + 0.95 for GPx indicating significant positive correlation with IOP. Similarly, at the mRNA level, an inverse correlation between TIMP gene expression and IOP (r = −0.93). The correlation was also significant for the genes; IL-1β (r = −0.83), MYOC (r = −0.84) and NFR2 (r = 0.93). Such correlation indicates that changes in biomarker abundance can impact biological processes such as aqueous humor dynamics and TM remodeling, shaping clinical responses. The observed molecular-clinical coupling supports a causal mechanistic pathway, highlighting the role of these biomarkers not only as indicators but also as drivers of physiological change in ocular hypertension. Furthermore, differences in correlation strength across treatment groups suggest distinct mechanistic actions, emphasizing the potential of biomarker-based stratification for optimizing treatment strategies. Such analysis emphasizes the importance of correlation as a tool for understanding disease mechanisms and guiding precision therapy.

**Table 4:** Correlation analysis between molecular data (protein expression by ELISA and fold change in gene expression) and clinical outcome (Change in IOP over time represented by AUC) between different rabbits (n = 5) receiving the optimized formula (LUT-EX@MN)

	Protein expression (ELISA)			Gene expression			
	TNF-α	IL-6	GPx	TIMP	IL-1β	MYOC	NFR2
r	−0.9398	−0.9630	0.9549	−0.9392	−0.8563	−0.84	0.93
r2	0.8832	0.9273	0.9119	0.8822	0.7332	0.70	0.86
p-value	0.0176	0.0085	0.0114	0.0178	0.0460	0.03	0.02

Correlation is represented by Pearson correlation coefficient (r), coefficient of determination (r^2^), and p-value

### Histological examination

**Fig. 6:**
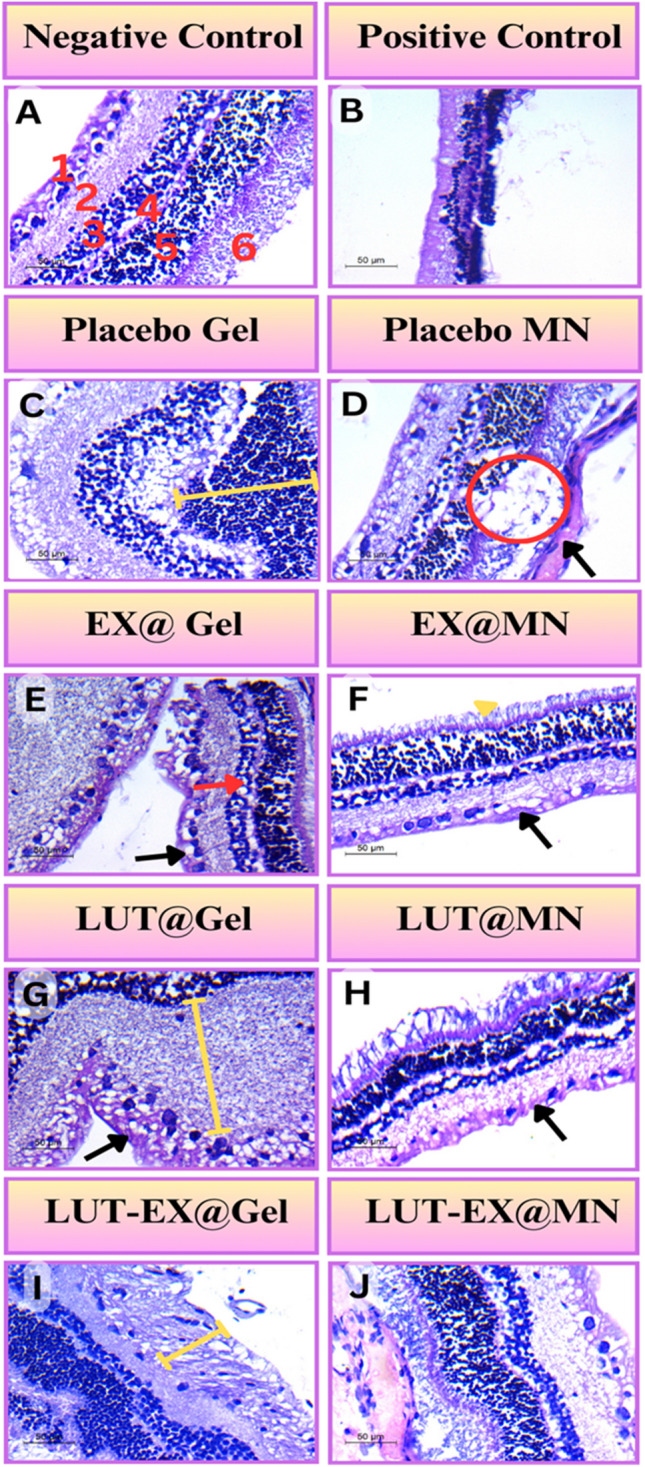
Photomicrographs of rabbits’ eyes examining the retina via H&E staining, Scale bar 50µm. **A**) Negative control consists of the normal healthy retinal layers (1) ganglion cell layer (GCL), (2) inner plexiform layer, (3) inner nuclear layer, (4) outer plexiform layer, (5) outer nuclear layer, and (6) rod and cone lamina; **B)** positive control confirmed the induction of glaucoma where it showed distinctive damage to the whole identity of retina layers. **C)** Placebo Gel group showed outer nuclear layer increased thickness (yellow line). **D)** Placebo MN group showed degenerated layers (red circle) and edema in GCL (black arrows). **E)** EX@ Gel group showed congestion in GCL layer (black arrow) and abnormal inner nuclear layer (red arrow). **F)** EX@MN group showed normal layer rods and cones layer (yellow triangle) with edema and congestion of GCL (black arrow). **G)** LUT@Gel group showed exaggerated inner plexiform layer (yellow line) and edema in GCL layer (black arrow). **H)** LUT@MN group comprises the normal layers with minimal apoptosis in GCL (black arrow). **I)** LUT-EX@Gel group showed abnormal layers specifically the GCL without distinctive neurons (yellow line). **J)** LUT-EX@MN group comprised near normal retina layers

The retina is composed of distinct neuron layers joined by synapses. In the negative control group, our findings showed normal observations of different cell types in the retina, including glial cells, photoreceptor cells (rods and cones) and neuronal cells (Fig. 6A).

In the positive control, the most common pathological abnormalities seen in the glaucomatous retina were reduced retinal thickness and substantial loss of RGCs. The responses of these glial cells are most likely directly related to neuronal degeneration in the glaucomatous retina. Furthermore, reduced thicknesses of, the inner nuclear layer (INL), the inner plexiform layer (IPL), and the outer nuclear layer (ONL) were also all observed (Fig. 6B).

When Placebo MN was compared to Placebo Gel, the results of Placebo MN demonstrated the spread of degeneration from the IPL to the outer nuclear layer, with observed edema in GCL. (Fig. 6D) Unlike this, the placebo gel showed a lower density of GCL and an uneven distribution of the outer nuclear layer. Overall, both groups showed modest progress from the positive control group (Fig. 6C).

When EX@MN and EX@gel were compared, the findings showed that EX@MN was significantly more effective than EX@gel. EX@MN showed normal rods and cones, with a better-organized retina layer, with some remaining congestion in the GCL (Fig. 6F). EX@gel group showed a major anomaly in the GCL with the inner nuclear layer (Fig. 6E).

Exchanging exosomes from previous groups with LUT indicated a modest synergistic impact of LUT with both MN and Gel, resulting in considerable expansion of the IPL and apoptosis in GCL in the LUT@Gel group (Fig. 6G). As a result, LUT@MN performed less well than EX@MN, as seen by the overall look of the layers (Fig. 6H). Overall, the LUT-EX@Gel group demonstrated significant disruptions in the GCL and IPL, as well as irregularities in the other layers (Fig. 6I). On the other hand, the LUT-EX@MN group exhibited the perfect layer distribution, resulting in considerable improvements when compared to the control group. It indicated a somewhat typical density of photoreceptor cells (rods and cones), neuronal cells, and glial cells (Fig. 6J).

## Conclusion

In conclusion, our study highlights the potential of using the naturally derived luteolin-loaded colostrum-derived exosomes (LUT-EX) and propolis integrated into microneedle (MN) arrays as a novel and effective approach for glaucoma treatment. The successful isolation and optimization of bovine colostrum-derived exosomes, combined with their effective incorporation into MN arrays, highlight the feasibility and safety of this approach. Their minimal invasiveness, low irritation and toxicity, enhanced permeability, and effective penetration of scleral tissues, combined with sustained intraocular pressure-lowering effects, circumvent the limitations of traditional IOP-lowering therapies, suggesting a promising therapeutic approach for glaucoma patients. Moreover, on the molecular level, LUT-EX@MN arrays reversed glaucoma-induced biochemical changes, such as the inflammatory markers; TNF-α, IL-8, and IL-1β, glaucoma-related biomarkers; MYOC, NRF2, and TIMP1 and the activity of glutathione peroxidase to levels comparable to healthy controls. This platform not only offers a promising alternative to conventional treatments but also paves the way for more precise and targeted ocular therapies, potentially revolutionizing the management of glaucoma and improving patient outcomes. Future research should focus on scaling up production, optimizing dosing regimens, and conducting clinical trials to confirm these findings and explore the broader applicability of this technology in ocular drug delivery.

## Supplementary Information

Below is the link to the electronic supplementary material.

Supplementary file1 (DOCX 205 KB)

## Data Availability

The authors confirm that the data for this study's findings are available within the article.

## Declarations

### Animal studies

Animal studies were performed following the regulations of the National Research Council’s Guide for the Care and Use of Laboratory Animals and approved by the Ethics Commission of Medical Research, Faculty of Pharmacy, Alexandria University Institute (ALEXU-IACUC AU-06–2023-11–11-1–206). Animals’ distress was reduced following the internationally accepted principles for laboratory use and care of the International Council of Laboratory Animal Science (ICLAS).
