# Correction: Microneedle loaded with luteolin-colostrum-derived exosomes: a dropless approach for treatment of glaucoma

**DOI:** 10.1007/s13346-026-02119-4

**Published:** 2026-04-09

**Authors:** Sarah A. Elsherbiny, Amal H. El-Kamel, Basant A. Bakr, Lamia A. Heikal

**Affiliations:** 1 Department of Pharmaceutics, Faculty of Pharmacy, Alexandria University, Alexandria, Egypt; 2 Department of Zoology, Faculty of Science, Alexandria University, Alexandria, Egypt

**Correction: Drug Delivery and Translational Research (2025) 16:613-634**

https://doi.org/10.1007/s13346-025-01914-9

In the original article there are errors in Fig. 4 and Table 2. Figure 4a includes duplicate photographs (saline and placebo). The sequence in the last row of Table 2 is incorrect. Following are the corrected figure and table:

**Fig. 4:**
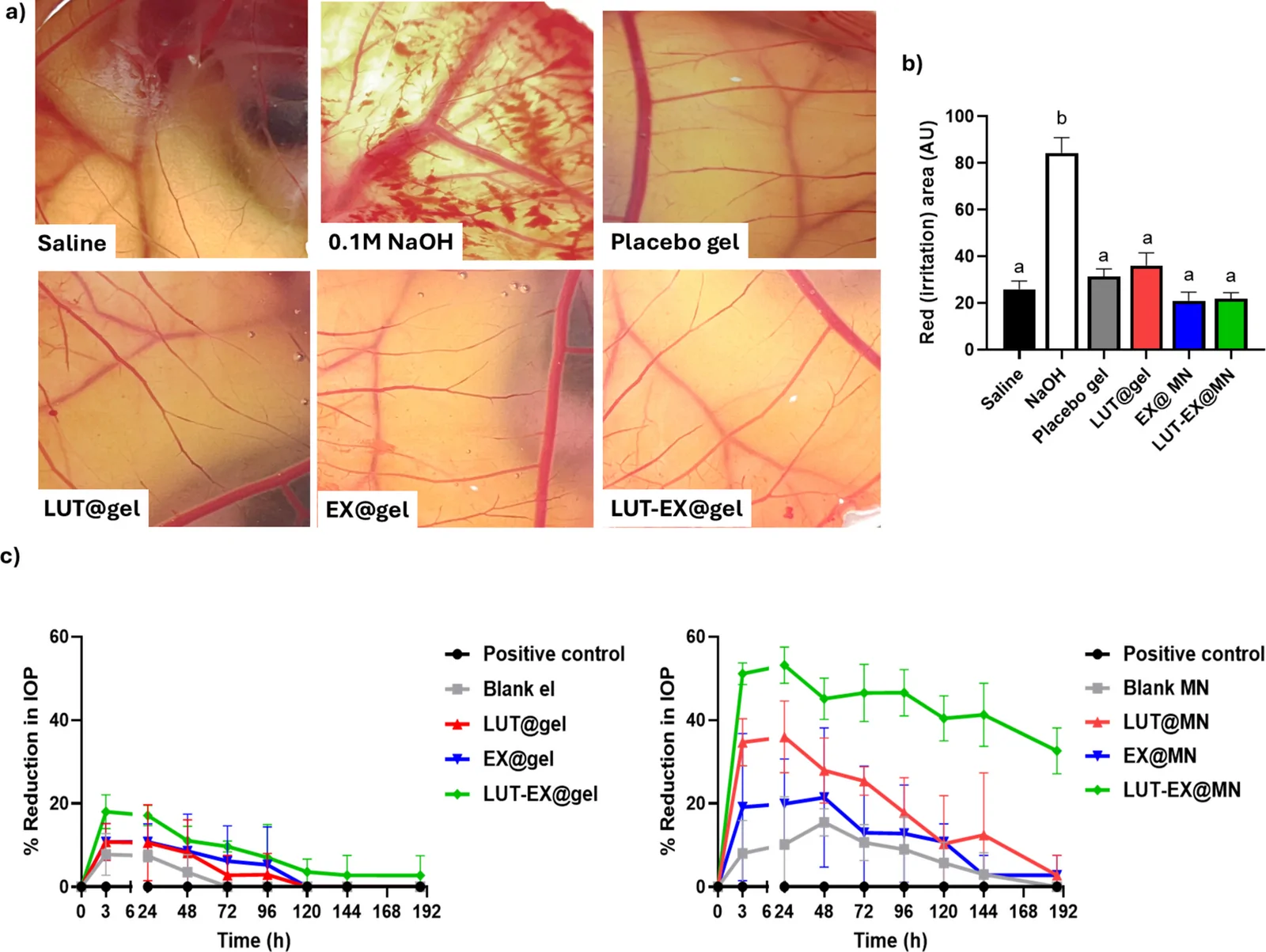
**a**) Images of chorioallantoic membrane test conducted on hen’s eggs (HET-CAM) following different treatments at room temperature for prediction of the potential of ocular discomfort and irritation potential. **b**) Scoring of ocular irritation in each sample via measuring the red irritated area and **c**) Percentage reduction in intraocular pressure following topical ocular insertion of a single dosage of different fabricated MN and their corresponding gel matrix over a period of seven days following glaucoma induction in rabbits

**Table 2:** Sequence of forward and reverse prime of rabbit myocilin (MYOC), Nuclear factor erythroid 2-Related Factor 2 (NRF2), tissue inhibitor of metalloproteinases (TIMP), IL-1β and GAPDH genes

	Forward	Reverse
Rabbit MYOC	CATCCGCCAGGTCTTTGAGT	CGTGTAGCCACCCCAAGAAT
Rabbit NRF2	TTCACATTTTCTGCCTCCGC	GTTTGGGAATGTGGGCAACC
Rabbit TIMP	CTTCTCTCAACGTTCCGGCT	GGATGCACAGGCAAACACTG
Rabbit IL-1β	GCACAACAGATCGCTTTGGG	TTCTCCAGAGCCACAACGAC
Rabbit GAPDH	AAGGGTCATCATCTCAGCCC	TGCGTTGCTGACAATCTTGAG

