# Supplementary material for: Microneedle loaded with luteolin-colostrum-derived exosomes: a dropless approach for treatment of glaucoma
Source: Drug Deliv Transl Res. 2025 Jul 11;16(2):613–34. doi: 10.1007/s13346-025-01914-9 (PMC12819496; doi:10.1007/s13346-025-01914-9)
Supplement: Supplementary file 1 — Supplementary file1 (DOCX 205 KB) [file 13346_2025_1914_MOESM1_ESM.docx]

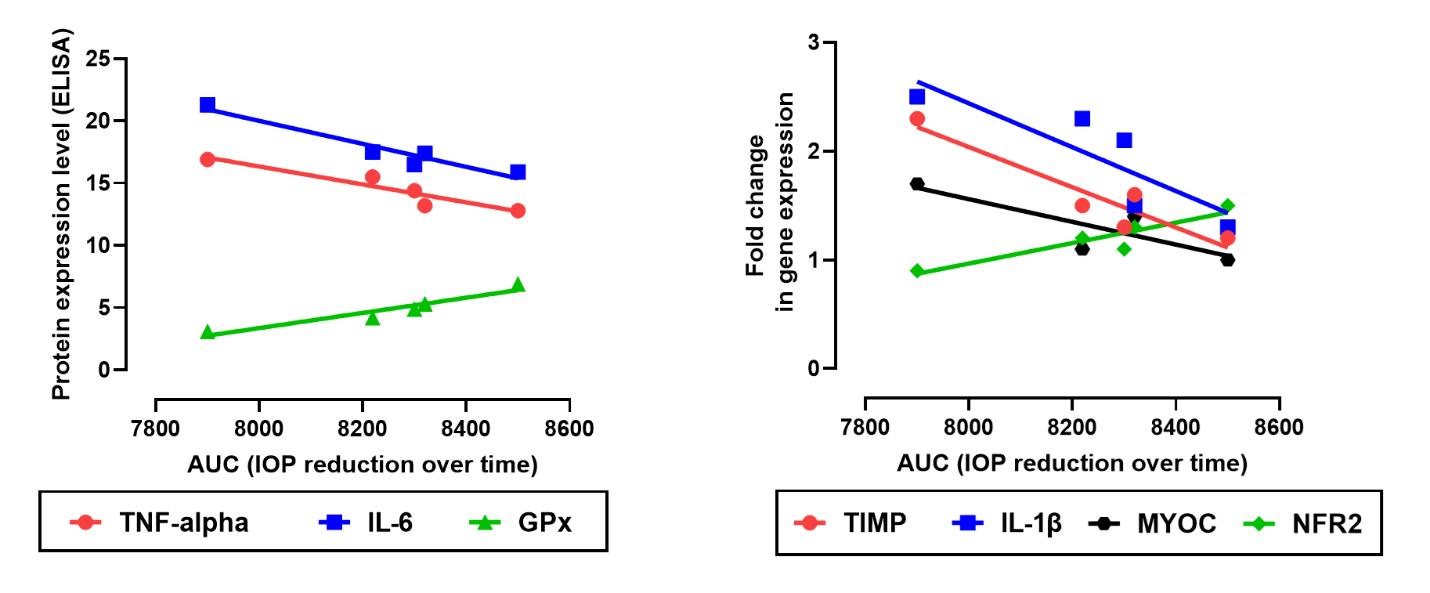
**Supplementary Figure**

**Figure S1: Correlation analysis between molecular data (a) protein expression by ELISA and (b) fold change in gene expression) and clinical outcome (change in IOP over time represented by AUC) for rabbits receiving the optimized formula (LUT-EX@MN), (n=5).**
